# Systematic evaluation of membrane-camouflaged nanoparticles in neutralizing *Clostridium perfringens* ε-toxin

**DOI:** 10.1186/s12951-023-01852-z

**Published:** 2023-03-17

**Authors:** Jinglin Xu, Dongxue Li, Lin Kang, Tingting Liu, Jing Huang, Jiaxin Li, Jing Lv, Jing Wang, Shan Gao, Yanwei Li, Bing Yuan, Baohua Zhao, Jinglin Wang, Wenwen Xin

**Affiliations:** 1 Hebei Key Laboratory of Animal Physiology, Biochemistry and Molecular Biology, College of Life Sciences, Hebei Normal University, Shijiazhuang, China; 2 State Key Laboratory of Pathogen and Biosecurity, Institute of Microbiology and Epidemiology, Academy of Military Medical Sciences (AMMS), Beijing, China

**Keywords:** Membrane-camouflaged, Nanoparticle, *Clostridium perfringens* ε-toxin, Red blood cell membrane, Intravenous injection, Pulmonary inhalation, Therapy

## Abstract

*Clostridium perfringens* ε-toxin (ETX) is the main toxin leading to enterotoxemia of sheep and goats and is classified as a potential biological weapon. In addition, no effective treatment drug is currently available in clinical practice for this toxin. We developed membrane-camouflaged nanoparticles (MNPs) with different membrane origins to neutralize ETX and protect the host from fatal ETX intoxication. We evaluated the safety and therapeutic efficacy of these MNPs in vitro and in vivo. Compared with membranes from karyocytes, such as Madin-Darby canine kidney (MDCK) cells and mouse neuroblastoma N2a cells (N2a cells), membrane from erythrocytes, which do not induce any immune response, are superior in safety. The protective ability of MNPs was evaluated by intravenous injection and lung delivery. We demonstrate that nebulized inhalation is as safe as intravenous injection and that both modalities can effectively protect mice against ETX. In particular, pulmonary delivery of nanoparticles more effectively treated the challenge of inhaled toxins than intravenously injected nanoparticles. Moreover, MNPs can alter the biological distribution of ETX among different organs in the body, and ETX was captured, neutralized and slowly delivered to the liver and spleen, where nanoparticles with ETX could be phagocytized and metabolized. This demonstrates how MNPs treat toxin infections in *vivo*. Finally, we injected the MNPs into mice in advance to find out whether MNPs can provide preventive protection, and the results showed that the long-cycle MNPs could provide at least a 3-day protection in mice. These findings demonstrate that MNPs provide safe and effective protection against ETX intoxication, provide new insights into membrane choices and delivery routes of nanoparticles, and new evidence of the ability of nanoparticles to provide preventive protection against infections.

**Graphical Abstract:**

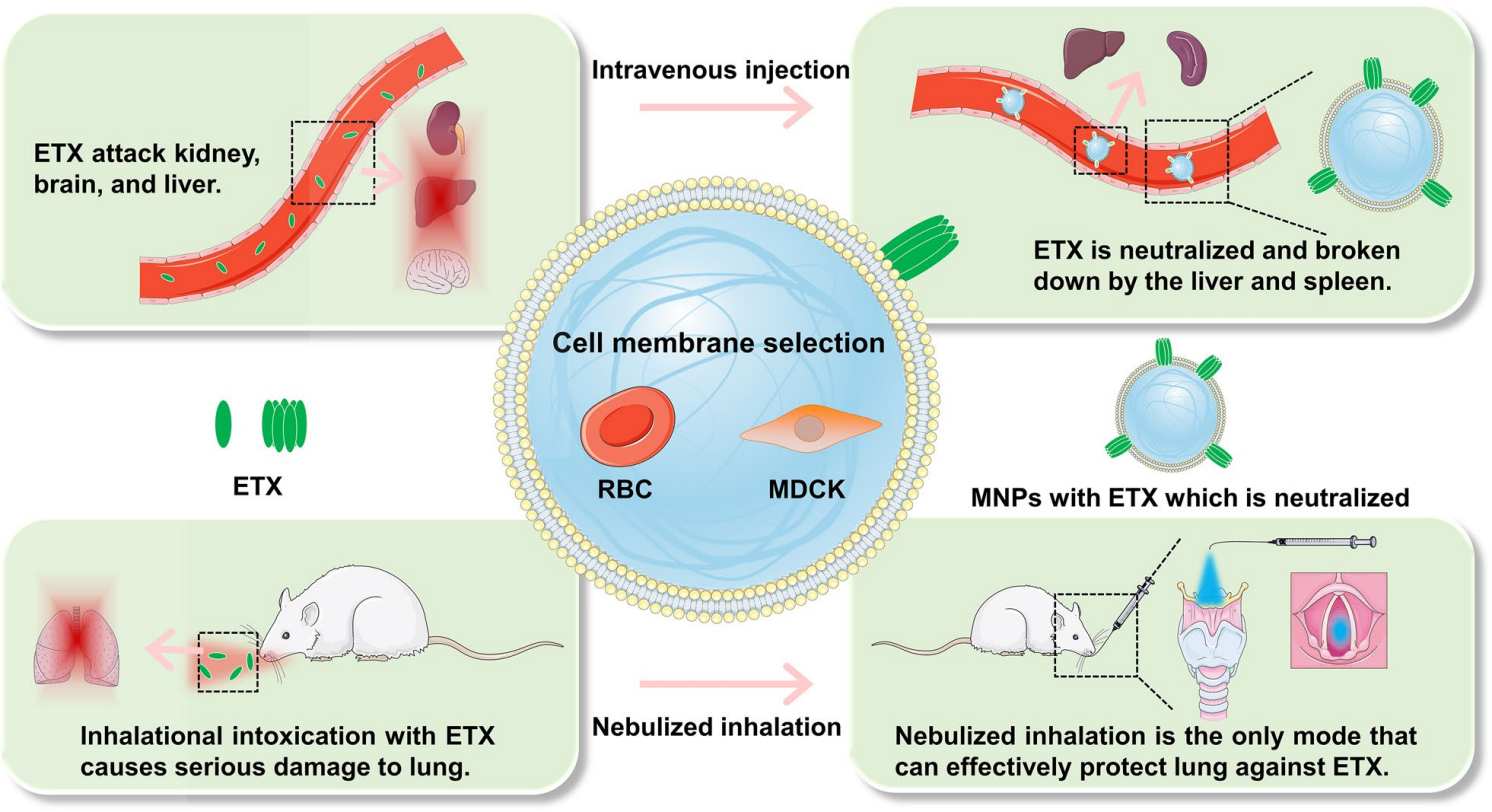

**Supplementary Information:**

The online version contains supplementary material available at 10.1186/s12951-023-01852-z.

## Introduction

The bacterium *Clostridium perfringens* produces a remarkable seventeen exotoxins, and of these, ε-toxin (ETX) is by far the deadliest [1, 2]. ETX is synthesized by *C. perfringens* types B and D. This toxin is the main virulence factor of *C. perfringens* type D, which is responsible for enterotoxemia in sheep, goat, and, more rarely, cattle. Enterotoxemia is a rapidly fatal disease that causes important economic losses globally [3]. ETX is the most powerful known toxin after botulinum and tetanus toxins [4]. Therefore, ETX is regarded as potential threat to national security and the animal husbandry economy. Moreover, ETX is classified as a potential biological weapon in some countries and as a category B biological agent by the Centers for Disease Control and Prevention (CDC) of the United States [5, 6]. The animal kidney is the primary target organ for ETX, followed by the brain [7]. ETX causes congestion and edema when target organs accumulate large quantities of toxin, leading to the death of animals [8]. Although there have been few reports of human infection with ETX [7, 9], our previous studies showed that ETX can form pores on human mature red blood cells (RBC) and produce hemolytic effects in a short period of time [10]. Therefore, the threat of ETX to human health is real, and as yet, there is no effective treatment drug in clinical practice. These features of ETX make it impossible for us to ignore its potential as a human threat. Therefore, developing a new safe and efficient drug to treat ETX infection is high priority.

In recent years, the rapid development of nanotechnology has extended the application of nanoproducts, especially in the field of medical treatment [11–17]. Nanoparticle-based methods of diagnosing and treating diseases have certain advantages in efficacy and safety compared to traditional methods [18, 19]. Among them are membrane-camouflaged biomimetic approaches [20] that use naturally-derived cell membranes to directly endow nanoparticles with enhanced biological interface capabilities [19]. Nanoparticles wrapped with cell membranes essentially replicate the properties of membrane-derived cells [21], allowing these membrane-camouflaged nanoparticles (MNPs) to treat various injuries and diseases caused by pore-forming toxins [22]. Current studies focus more on nanoparticle design and fabrication, with limited evaluation of membrane choice, delivery route, and mechanism of detoxification, all of which are important for application of MNPs.

**Fig. 1:**
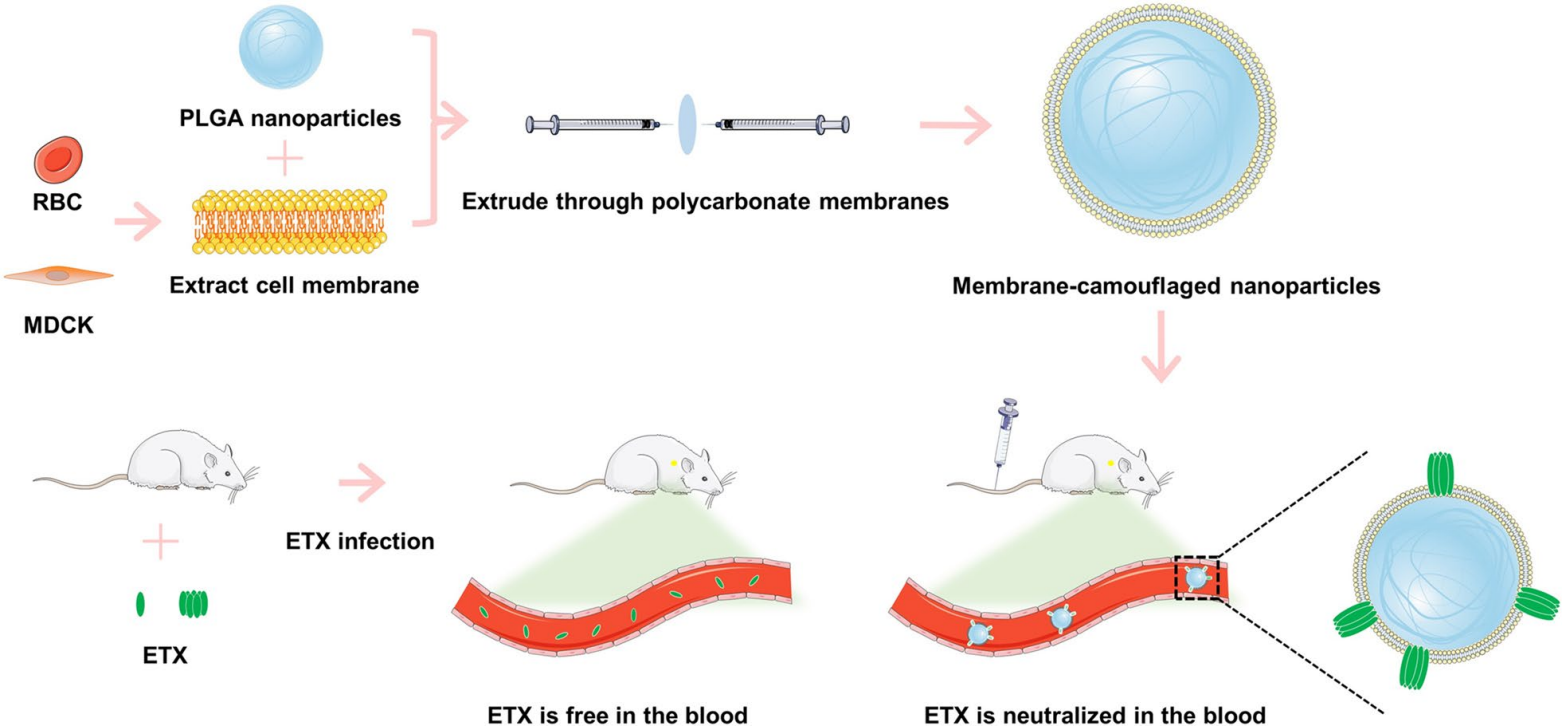
Schematic illustration of the procedure used to prepare RBC membrane-camouflaged nanoparticles (RBC-NPs) or MDCK cell membrane-camouflaged nanoparticles (MDCK-NPs). Membrane-camouflaged NPs can neutralize ETX in mice infected with ETX

Given that ETX is a pore-forming toxin, we hypothesized that MNPs might be used to neutralize ETX. MDCK cells are highly sensitive to ETX [23, 24], and ETX is able to form pores on the membrane of red blood cells (RBCs) [10]. Thus, MDCK cells and RBCs were used as materials to prepare MNPs for neutralizing ETX. In this study, we designed two MNP types using different membranes to neutralize ETX, and systematically evaluated the safety and neutralization ability of these MNPs in vitro and in vivo. MNPs were fabricated by mechanically squeezing the extracted MDCK cell membranes and RBC membranes tightly onto the nanoparticles, as previously described [20] (Fig. 1). The MNPs were able to neutralize ETX in the blood of mice infected with ETX, reducing symptoms of poisoning and successfully treating mice (Fig. 1).

## Results

### Packaging of nanoparticles into cell membranes and characterization of MNPs

**Fig. 2:**
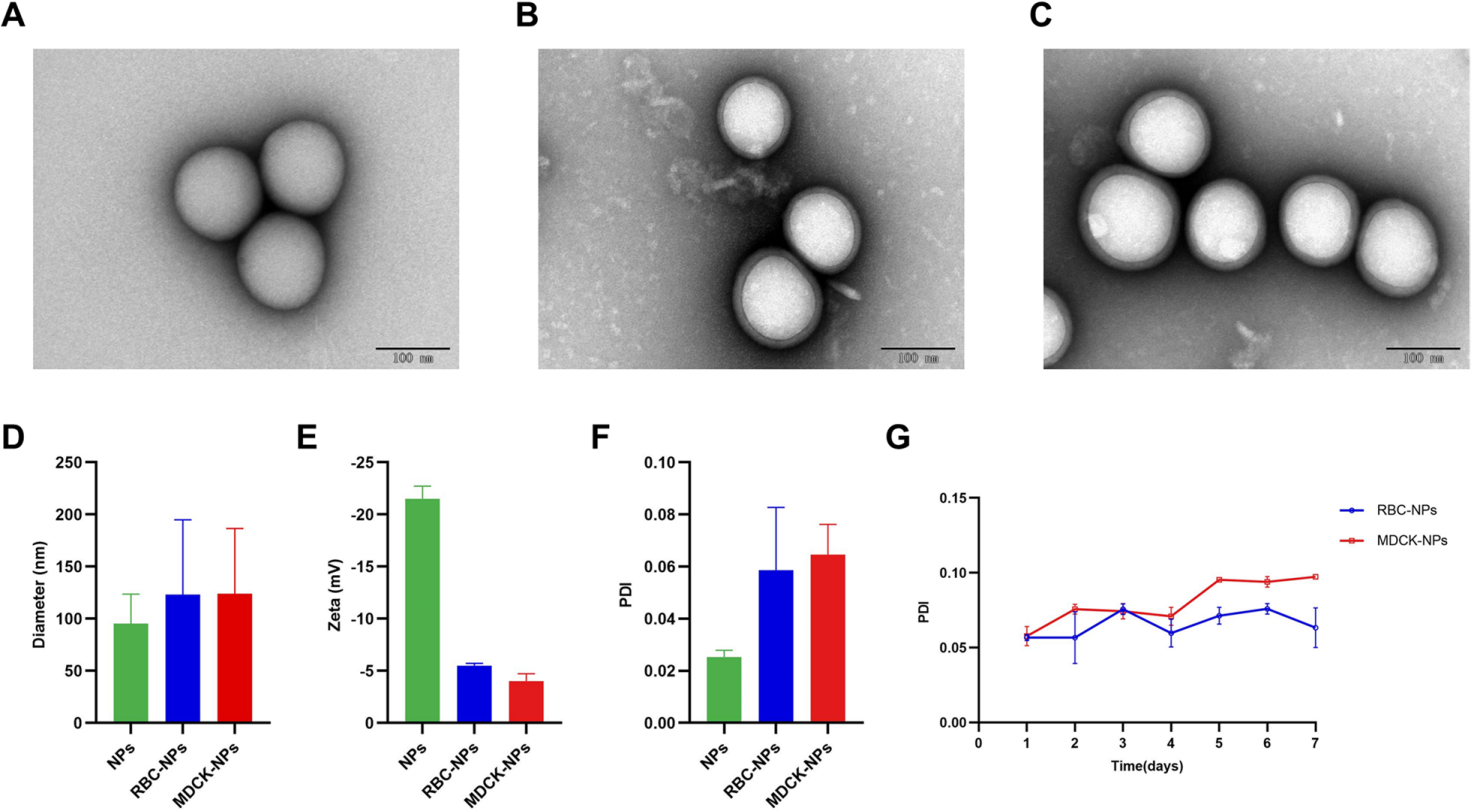
Characterization of nanoparticles. TEM images of **A** bare NPs, **B** RBC-NPs, and **C** MDCK-NPs. **D** Flow NanoAnalyzer measures of nanoparticle diameter (nm). **E** DLS measurements of zeta potential (Zeta, mV) of nanoparticles. **F** DLS measurements of PDI of nanoparticles. **G** DLS measurements of the changes in the PDI of the MNPs in PBS at 37 °C. Data are presented as the means ± SD

We prepared the 50:50 Poly (DL-lactide-co-glycolide) Carboxylate End Group (PLGA) nanoparticles, co-incubated them with purified cell membranes, and extruded the result from polycarbonate membranes to generate cell-membrane coated PLGA nanoparticles (Fig. 1). Transmission electron microscopy (TEM) was used to observe three types of nanoparticles: bare nanoparticles (bare NPs), coated with RBC cell membrane (RBC-NPs), and coated with MDCK cell membrane (MDCK-NPs) (Fig. 2A–C). All nanoparticles exhibited spherical structures. When RBC-NPs and MDCK-NPs are compared to bare NPs, it can be clearly seen that the surface is covered with a monolayer film. A Flow NanoAnalyzer was used to measure the diameter (DH) of the three kinds of nanoparticles; bare NPs were ~ 95 nm, and RBC-NPs and MDCK-NPs were ~ 123 nm and ~ 124 nm, respectively, or ~ 30 nm larger than bare NPs (Fig. 2D). Zeta potentials of the three nanoparticles measured using dynamic light scattering (DLS), indicated that the zeta potentials of RBC-NPs and MDCK-NPs were ~ 15 mV greater than bare NPs (Fig. 2E). To assess the dispersion of nanoparticles in liquids, the polydispersity index (PDI) of nanoparticles dispersed in PBS was measured; PDI was less than 0.2, indicating that the three nanoparticles have good dispersion (Fig. 2F). No change in PDI a week later indicated that nanoparticles can be stably dispersed in PBS over the short term (Fig. 2G).

### In vitro evaluations of MNPs

**Fig. 3:**
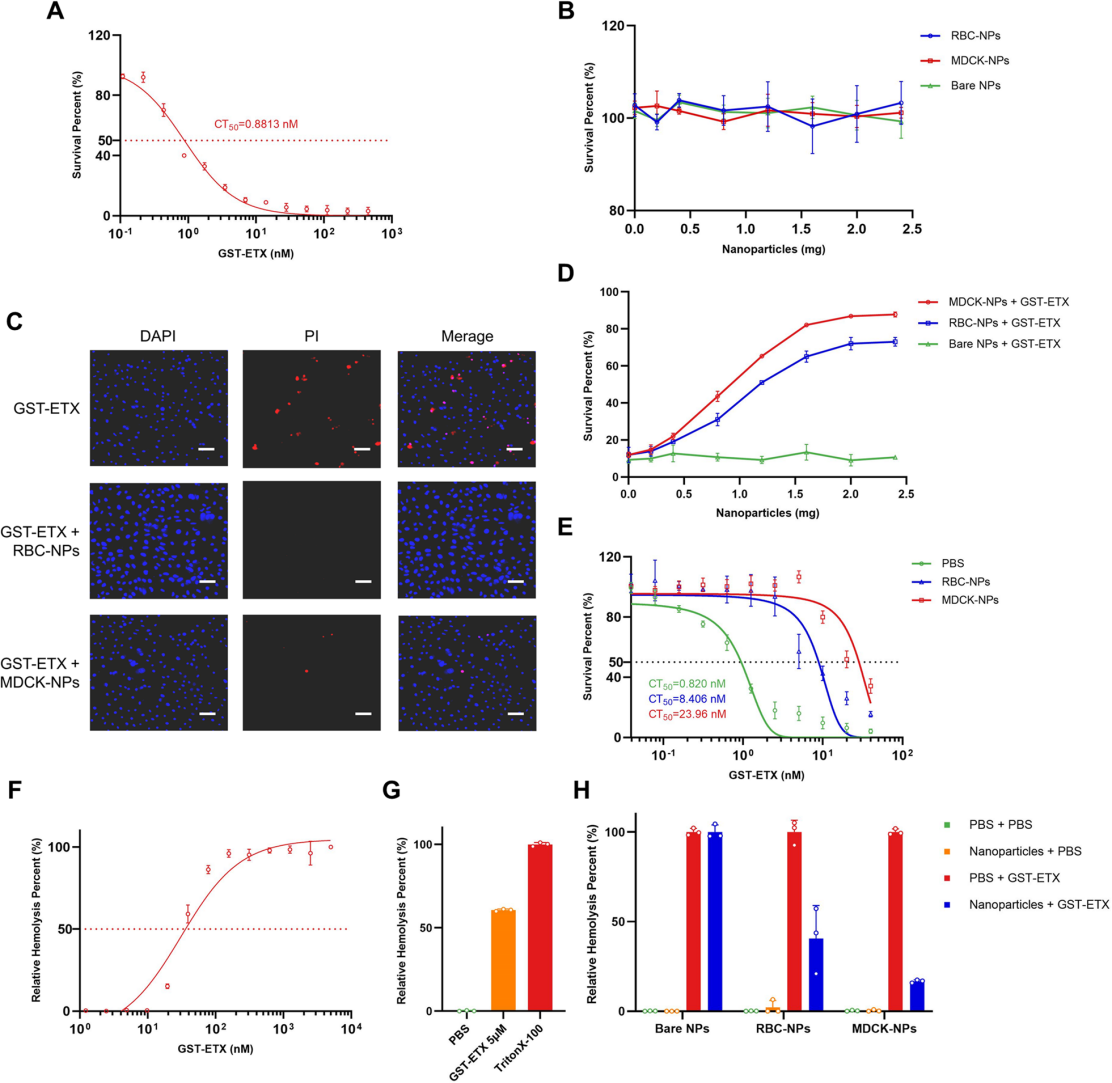
In vitro evaluation of GST-ETX and MNPs. **A** *In vitr*o toxicities of GST-ETX. CT_50_ values were calculated based on concentration–survival curves. **B** MDCK cells were exposed to increasing concentrations of nanoparticles for 1 h at 37 °C and survival of MDCK cells measured. **C** Cells were observed by confocal microscopy. (Scale bar: 100 μm). **D** MDCK cells were exposed to increasing concentrations of nanoparticles and 20 nM of GST-ETX for 1 h at 37 °C. Survival of MDCK cells was measured. **E** A series of concentrations of GST-ETX were treated with two kinds of nanoparticles or PBS for 1 h at 37 °C. Toxicities of the mixtures were measured. **F** RBCs in 2.5% solution were incubated with increasing concentrations of GST-ETX for 1 h at 37 °C. Relative hemolysis of RBCs was measured. **G** RBCs in 2.5% solution were incubated with GST-ETX (5 μM) or 1% TritonX-100 for 1 h at 37 °C. Hemolysis of RBCs caused by Triton X-100 is defined as 100%. Maximal extent of hemolysis induced by GST-ETX was measured. **H** RBCs in 2.5% solution were incubated with GST-ETX (30 nM) and nanoparticles for 1 h at 37 °C. An assay of relative hemolysis of RBCs was performed. Data are presented as mean ± SD

Neutralization capacity in vitro is an important indicator of in vivo therapeutic efficacy. We therefore assessed the neutralization capacity of the two kinds of MNPs (RBC-NPs and MDCK-NPs) to recombinant ETX with Glutathione-S-transferase (GST) tags (GST-ETX) in vitro by 3-(4,5-Dimethylthiazol-2-yl)-5(3-carboxymethoxyphenyl)-2-(4-sulfophenyl)-2H-tetrazolium inner salt (MTS) assay with cells. MDCK cells are the most sensitive cells to ETX and are commonly used in ETX cytotoxicity. ETX with different tags (GST and 6 × His) did not significantly differ in toxicities (Additional file 1: Fig. S1). Therefore, MDCK cells were used in all evaluations of GST-ETX and MNPs in vitro. The concentration of GST-ETX that killed 50% of MDCK cells (denoted by CT_50_) was 0.8813 nM (Fig. 3A). When the toxin reached a concentration of 20 nM, maximum cell mortality was achieved. To investigate whether the three kinds of nanoparticles could damage MDCK cells, MDCK cells were treated with a series of concentrations of nanoparticles. A measure of nanoparticles as high as 2.4 mg showed no cytotoxicity to MDCK cells (Fig. 3B).

Cell death was examined using a confocal high-content imaging system (Fig. 3C, Additional file 1: Fig. S2). MDCK cells incubated with the two kinds of MNPs maintained a high survival rate under ETX challenge. MDCK cells were incubated with GST-ETX and a series of concentrations of MNPs. MNPs significantly reduced the toxicity of GST-ETX in a dose-dependent manner, with 2 mg MNPs nearly abolishing the toxicity of GST-ETX to MDCK cells (Fig. 3D). MDCK-NPs reduced the toxicity of GST-ETX more than RBC-NPs, suggesting that MDCK-NPs can neutralize GST-ETX faster. We next treated a series of concentrations of GST-ETX with MNPs, to assess the neutralization ability of MNPs mixed with toxin. Both MNPs neutralized ETX, reducing the toxicity of the mixture to MDCK cells (Fig. 3E). The two types of MNPs created mixtures that differed in CT_50_, with 2 mg of RBC-NPs neutralizing 3.793 pmol GST-ETX, and 2 mg of MDCK-NPs neutralizing 11.75 pmol GST-ETX. Thus, while both kinds of MNPs can effectively neutralize ETX, the MDCK-NPs neutralized more GST-ETX faster when cells were challenged by GST-ETX. Therefore, MDCK-NPs have a better performance against GST-ETX than RBC-NPs.

To verify the difference in sensitivity between RBCs and MDCK cells, we assessed the relative hemolysis damage in RBCs caused by GST-ETX. Hemolysis increased with the concentration of GST-ETX (Fig. 3F). The concentration of GST-ETX required to cause hemolysis in RBCs is close to 20 nM, and the maximum relative hemolysis was achieved at a toxin concentration of approximately 150 nM. In addition, RBC hemolysis by GST-ETX was not complete within 1 h (Fig. 3G). In contrast, 20 nM GST-ETX caused maximum cell mortality to MDCK cells within 1 h. This indicates that RBCs and MDCK cells differ in sensitivity to ETX, and this difference is reflected in the differences between the two kinds of MNPs. We estimated the concentration of 30 nM of GST-ETX that would cause 50% relative hemolysis to verify the neutralization capacity of MNPs. We calculated that a complete neutralization of 30 nM GST-ETX 1 ml would require about 5.1 mg of MDCK-NPs and about 15.8 mg of RBC-NPs. The results of co-incubation with RBCs showed that MDCK-NPs could reduce the toxicity by 85%, and RBC-NPs could reduce the toxicity by 60% (Fig. 3H). None of the three kinds of nanoparticles caused any damage to RBCs. This result is consistent with the difference in protective ability of the two MNPs in the MDCK cell protection experiment above. When MNPs and cells competitively bind to GST-ETX, MDCK-NPs can neutralize more toxin faster. This may be because there are more receptors on the membranes of MDCK cells, given that MDCK cells are more sensitive than RBCs to GST-ETX.

### In vivo safety assessment of MNPs

**Fig. 4:**
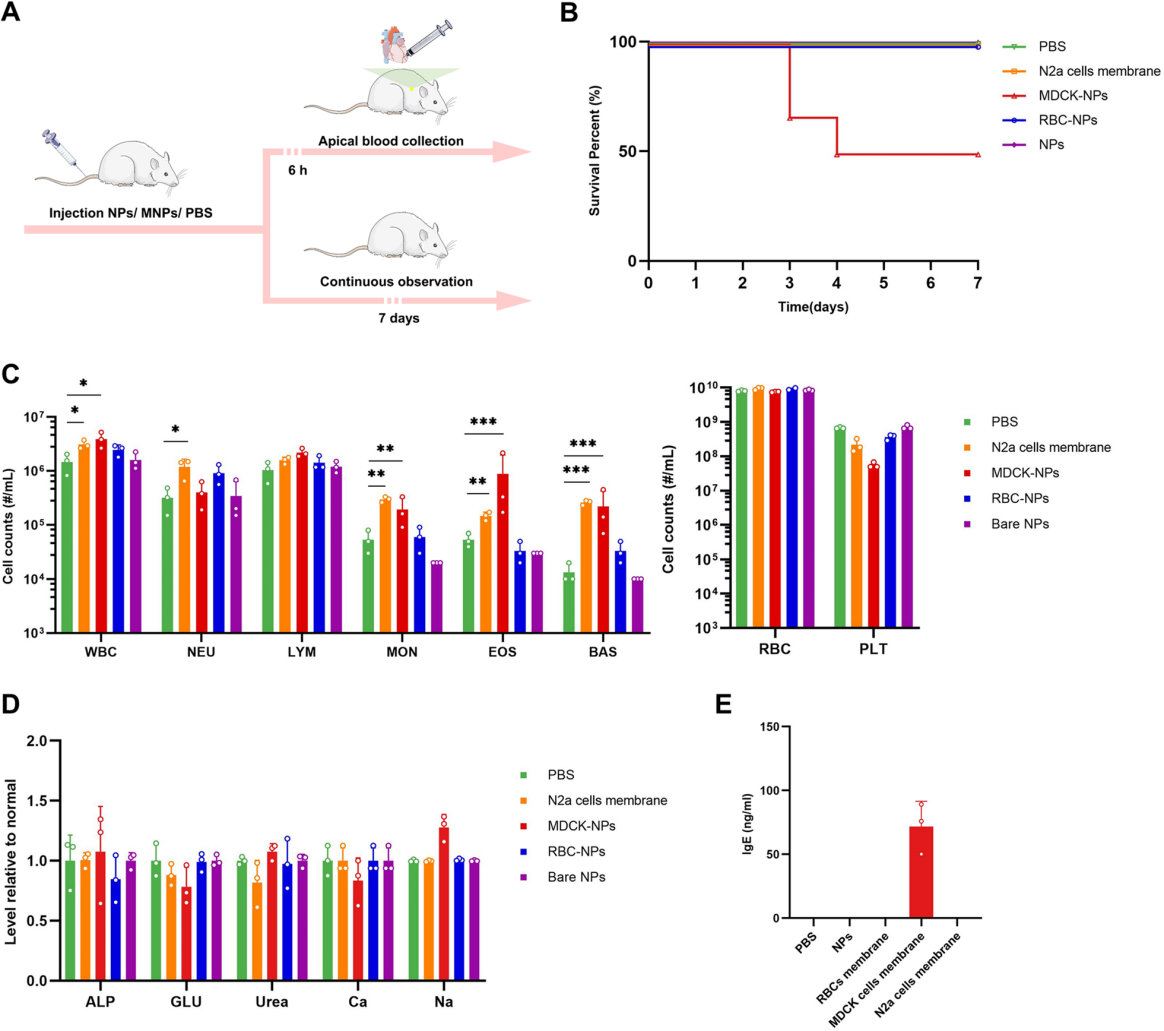
In vivo safety evaluations of the three kinds of the nanoparticles. **A** Schematic illustration of the experiments. Four groups of 8-week-old female BALB/c mice were injected with 100 μL PBS or PBS with bare NPs, with RBC-NPs or with MDCK-NPs (2% w/v) intravenously. **B** Survival curves of the mice in the next 7 days (n = 6). Five groups of 8-week-old female BALB/c mice, were injected intravenously with 100 μL PBS or PBS with nanoparticles (2% w/v) or N2a cell membrane (100 μL, approximately 5 × 10.^7^ cells). **C** Blood cell counts of the mice (n = 3). **D** Chemical composition analysis of serum (n = 3). **E** The concentration of IgE in plasma in the mice (n = 3). Data are presented as the mean ± SD. *p* < 0.05 (*), *p* < 0.01 (**), *p* < 0.001 (***), *p* ≥ 0.05 (ns)

Safety assessment in vivo is an important precondition of in vivo therapeutic efficacy. We therefore assessed the safety of the three kinds of nanoparticles to mice in vivo. Following injection of one of the three kinds of the nanoparticles to three groups of mice intravenously, mice were observed for 7 consecutive days (Fig. 4A). Bare NPs and RBC-NPs were safe to animals, while MDCK-NPs caused death in half of the mice (Fig. 4B). We examined multiple blood parameters, including a comprehensive serum chemistry panel and blood cell counts. Cells count in mice injected with bare NPs and RBC-NPs were consistent with baseline levels, indicating these two nanoparticles did not cause any toxic or immune response (Fig. 4C). In contrast, mice injected with MDCK-NPs, displayed significant anomalies in white blood cell (WBC), eosinophil (EOS), basophil (BAS) and platelet (PLT) counts, indicating that MDCK cell membranes could trigger an immune response, which is presumably related to MDCK-NP-induced death in mice. In addition, we assessed blood markers of liver and kidney damage; alkaline phosphatase (ALP) [25] and glucose (GLU) in the blood are thought to reflect damage to the liver [26], while serum calcium (Ca), serum sodium (Na), and urea in blood are thought to reflect damage to the kidney [27–31]. However, these indicators were normal in all mice (Fig. 4D), indicating the short-term safety of MNPs.

Immune response triggered by MDCK-NPs, which include increased EOS, BAS and decreased PLT, are presumed to be related to hypersensitivity [32–34]. To verify whether the anomalies of MDCK-NPs were related to hypersensitivity, we measured IgE in mouse serum. The results of serum IgE detection showed that IgE in the serum of mice injected with MDCK cells membrane was significantly increased (Fig. 4E), while no obvious IgE reaction was detected in the serum of the other mice. These results suggest that MDCK cell membranes induced strong hypersensitivity in mice, which is likely the main cause of death in mice injected with MDCK-NPs. MDCK cells originate from normal kidney cells of a cocker spaniel dog [35], which is distantly related to murine species, and we infer that the injection of membranes from a distantly related species are likely to induce strong immune response and hypersensitivity. To test our hypothesis, we injected membranes from N2a cells, which originate from a murine species. Membrane from N2a cells induced slight immune response but did not trigger hypersensitivity, in keeping with our hypothesis.

Given the unstable safety profile of MDCK-NPs, we abandoned the use of MDCK-NPs in further animal experiments, despite their excellent ability to compete and bind ETX.

### In vivo therapeutic efficacy evaluations of MNPs

**Fig. 5:**
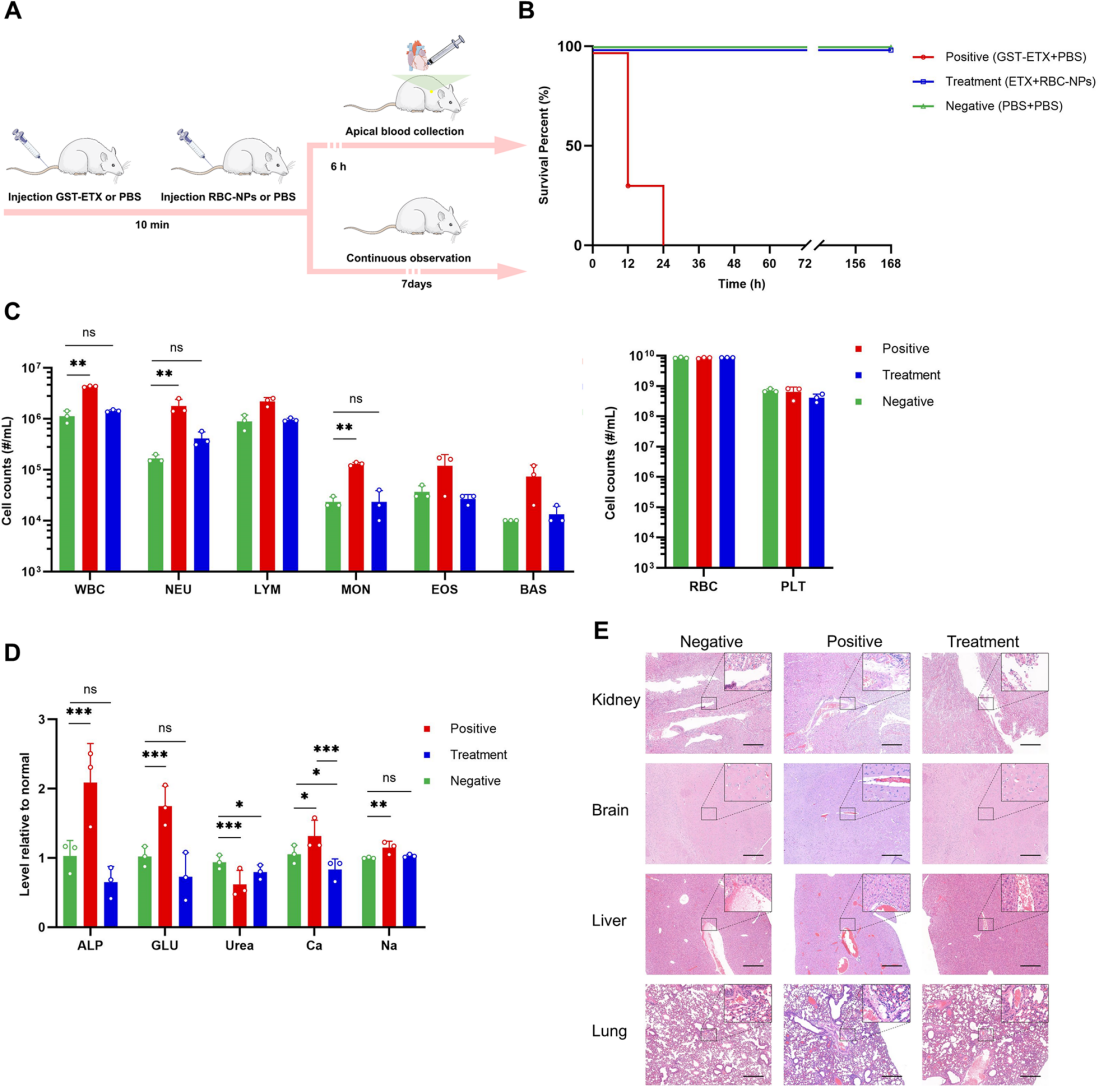
*In v*ivo therapeutic efficacy evaluations of the RBC-NPs. **A** Schematic illustration of the experiments. Three groups of mice were injected with 20 ng GST-ETX intravenously; 10 min later, mice in the treatment group were injected with 2 mg RBC-NPs intravenously and mice in the positive control group were injected with PBS intravenously. At the same time, mice in the negative control group were injected both times with PBS intravenously. **B** The survival curves of mice in the 7 days post-injection (n = 6). **C** Blood cell counts in the mice (n = 3). **D** Chemical composition analysis of serum (n = 3). **E** Major organs were stained with hematoxylin and eosin (H&E). Representative sections are shown for various organs of the mice in different groups (scale bar: 200 μm). Data are presented as mean ± SD. *p* < 0.05 (*), *p* < 0.01 (**), *p* < 0.001 (***), *p* ≥ 0.05 (ns)

After assessing neutralization capacity in vitro and the safety in vivo, we next assessed therapeutic efficacy of RBC-NPs in vivo. We first intravenously injected GST-ETX into mice of each group, and then 10 min later, we intravenously injected RBC-NPs or PBS into mice of the treatment and positive control group, respectively (Fig. 5A). The dose of 20 ng GST-ETX led to 100% death of mice in the positive control group within 24 h (Additional file 1: Fig. S3), while mice given RBC-NPs had 100% survival for 7 days (Fig. 5B). Histopathological analysis showed that the kidneys, brains, livers and lungs of mice in the positive control group had different degrees of damage, mainly manifested as congestion or edema; however, these symptoms were significantly relieved by RBC-NPs (Fig. 5C).

In another three groups of mice, 6 h after completion of the two injections, blood for analysis was done (Fig. 5A). Blood biochemical analysis revealed that, compared to the negative control group, WBC count in the blood of ETX-infected mice in the positive control group was significantly elevated 300%, and the change of WBC was caused mainly by 900% elevation of neutrophils (NEU) (Fig. 5D). WBC count in the blood of mice treated with RBC-NPs was closer to that of the negative control group of mice. This indicates that RBC-NPs effectively attenuated the inflammatory response triggered in vivo by the ETX challenge [36]. In addition, we assessed blood markers of liver and kidney damage. The livers and kidneys of mice were damaged to varying degrees during the ETX challenge, and RBC-NPs can play a role in protecting these organs, which was consistent with the results of histopathology analysis (Fig. 5E, Additional file 1: Fig. S4). Thus, RBC-NPs were sufficiently able to competitively bind ETX in vivo to protect host organs from damage and can effectively treat GST-ETX infection in vivo.

### The interaction between RBC-NPs and ETX and its metabolism in vivo

**Fig. 6:**
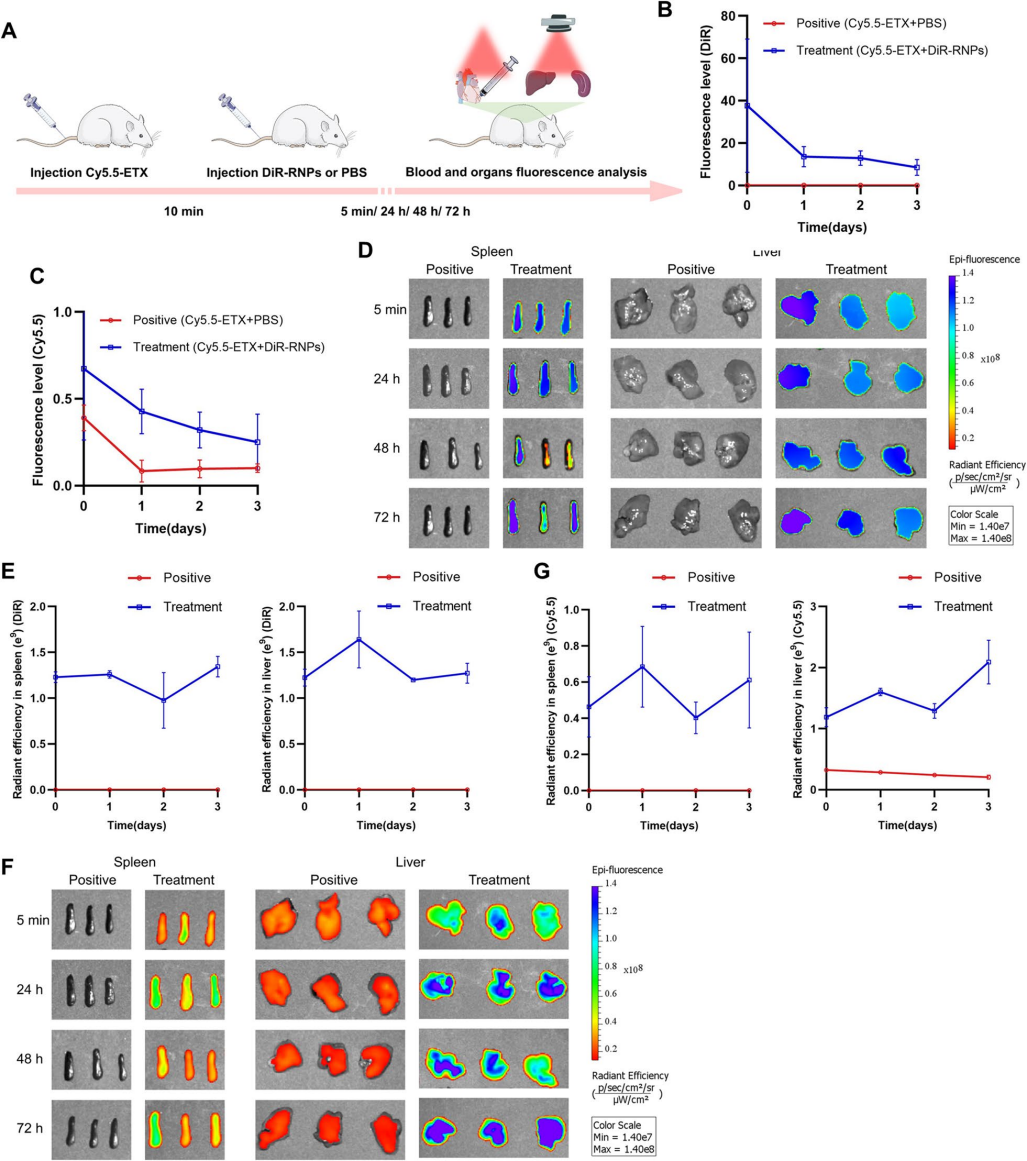
Interaction and metabolism between RBC-NPs and GST-ETX in vivo. **A** Schematic illustration of the experiments. Two groups of mice were injected with 5 ng GST-ETX intravenously, and 10 min later, mice in the treatment group were injected with 2 mg DiR-RNPs intravenously and mice in the positive control group were injected with PBS intravenously. Blood and organs were analyzed at 5 min, 24 h, 48 h and 72 h in vitro. **B** The fluorescence level of the DiR in blood (n = 3). **C** The fluorescence level of Cy5.5 in blood (*n* = 3). **D** In vitro fluorescence images of DiR in liver and spleen, **E** radiant efficiency of the DiR in liver and spleen (*n* = 3). **F** In vitro fluorescence images of Cy5.5 in liver and spleen. **G** Radiant efficiency of Cy5.5 in liver and spleen (*n* = 3). The positive control group was injected with Cy5.5-ETX and PBS, the treatment group was injected with Cy5.5-ETX and DiR-NPs. Data are presented as the mean ± SD

We further investigated the interaction between in metabolisms of RBC-NPs and GST-ETX in vivo to verify how the RBC-NPs protect the host. We injected mice intravenously with fluorescently-labeled RBC-NPs and GST-ETX, and the distribution of radiant efficiency in blood and tissues reflected their interactions and metabolisms. To display the metabolism of ETX toxin and RBC-NPs in mouse tissues, the NIR dye DiOC18(7) (DiR) was used to label RBC-NPs (DiR-RNPs), the NIR dye cyanine 5.5 (Cy5.5) was used to label GST-ETX (Cy5.5-ETX). We intravenously injected Cy5.5-ETX into mice of each group and 10 min later, intravenously injected DiR-RNPs or PBS into mice of the treatment group and the positive control group, respectively. At several time points post-injection (after 5 min, 24 h, 48 h and 72 h), blood of random mice in each group was taken for quantitative analysis of fluorescence intensities and its major organs were taken at the same time for fluorescence images in vitro (Fig. 6A). The quantitative analysis of fluorescence intensities in blood showed that DiR-RNPs in the blood of mice in the treatment group decreased over time (Fig. 6B). It should be noted that Cy5.5-ETX in the blood of mice in the treatment group was not completely cleared after three days, but consistently decreased along with DiR-RNPs (Fig. 6C). Levels of Cy5.5-ETX in the blood of mice in the treatment group was lower than that of the positive control group all time points and was reduced to a very low level 24 h after injection. Thus, within 5 min of injection, RBC-NPs neutralized the majority ETX, preventing ETX from spreading in vivo. In addition, the RBC-NPs that neutralized ETX remained stable in the blood and did not release toxins into the blood again.

Quantitative data of fluorescence images in vitro of DiR in tissues indicated that DiR-RNPs were captured by the liver and spleen (Fig. 6D–E, Additional file 1: Figs. S5, S6). The Cy5.5 signal of mice in the positive control group suggested Cy5.5-ETX gradually decreased in the liver, and no fluorescence signal was observed in the spleen (Fig. 6F–G, Additional file 1: Figs. S7, S8). In the treatment group, Cy5.5-ETX was much higher in the liver and also had a high signal in the spleen. As with the fluorescence signal in blood of the treatment group, the distribution signal of GST-ETX in organs is consistent with that of RBC-NPs. This was especially evident in the spleen, in which GST-ETX was not detected in the spleen in the positive control group, but because RBC-NPs had been captured by the spleen, it was detected in the spleen of the treatment group. The difference is that the two fluorescence signals tend to decrease over time in the blood, while fluorescent signals in the spleen and liver show a tendency to stabilize or increase. The results show that as immune and detoxification organs of animals, the spleen and liver captured RBC-NPs that had neutralized GST-ETX from the blood. This is how RBC-NPs treat GST-ETX infection in vivo.

### In vivo therapeutic efficacy evaluations of nebulized pulmonary inhalation

**Fig. 7:**
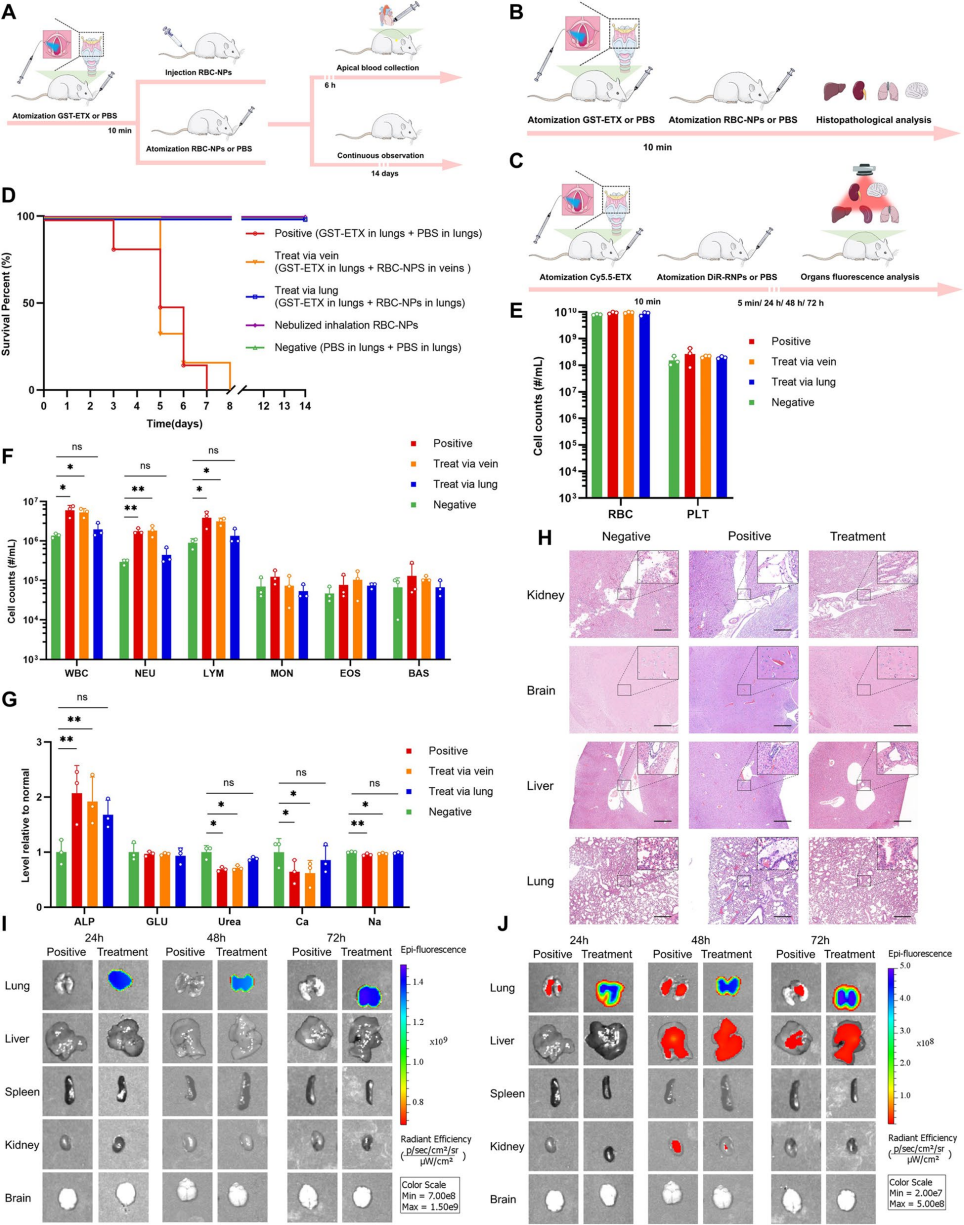
The in vivo therapeutic efficacy of RBC-NPs and metabolism of RBC-NPs and GST-ETX in lung. **A** Schematic illustration of the experiment. Four groups of mice were administered 50 ng GST-ETX via trachea; 10 min later, two treatment groups of mice were administered 2 mg RBC-NPs via trachea or intravenous, and a positive control group of mice was administered PBS via trachea. A negative control group of mice were administered PBS via trachea twice. **B** Schematic illustration of the experiment. Three groups of mice were administered 50 ng GST-ETX via trachea, 10 min later, treatment groups of mice were administered 2 mg RBC-NPs via trachea, and a positive control group of mice were administered PBS from tracheas. At the same time, a negative control group of mice were administered PBS via trachea twice. **C** Schematic illustration of metabolism experiment. Two groups of mice were administered 12.5 ng Cy5.5-ETX via trachea; 10 min later, the treatment group of mice were administered 2 mg DiR-RNPs via trachea, and the positive control group of mice were administered PBS from trachea. **D** Survival curves of mice over 14 days post-infection (n = 6). **E**, **F** Blood cell counts in mice (n = 3). **G** The chemical composition analysis of serum (n = 3). **H** Representative sections made from various organs of experimental mice, stained with H&E (scale bar: 200 μm). **I** In vitro fluorescence images of DiR in liver and spleen. **J** In vitro fluorescence images of Cy5.5 in liver and spleen. Data are presented as mean ± SD. *p* < 0.05 (*), *p* < 0.01 (**), *p* < 0.001 (***), *p* ≥ 0.05 (ns)

Toxic aerosols can be used as weapons in terrorist attacks. For ETX, which was classified as a potential biological weapon, whether RBC-NPs can play a protective role in ETX challenges from pulmonary inhalation is of key import. We therefore assessed the therapeutic efficacy of the RBC-NPs during nebulized pulmonary inhalation of mice in vivo. Liquid aerosol devices were used to administer GST-ETX and RBC-NPs from the mouse trachea by quantitative nebulization, thereby simulating ETX aerosol challenges and lung drug delivery. The advantage of using liquid aerosol devices is that the drug can be evenly distributed into the lungs of mice (Additional file 1: Fig. S9) and ensures that each mouse receives the drug at the same distribution location and reduces the risk of pulmonary edema caused by lung administration. This not only allows us to simulate an ETX aerosol weapons challenge, but also minimizes possible adverse effects during lung delivery of RBC-NPs. We first administered 50 ng GST-ETX into the tracheas of mice in each group, and 10 min later, administered 2 mg RBC-NPs by trachea or intravenously to mice in the two treatment groups; PBS was administered by trachea to mice in the positive control group. Mice in the negative control group had PBS administered by tracheas each time (Fig. 7A). Mice were observed for 14 days post-infection. Introduction of 50 ng GST-ETX into mouse lungs resulted in 100% mortality of the mice in the positive control group within 8 days (Additional file 1: Fig. S10). Treatment mice that had RBC-NPs introduced by aerosol into the lungs had 100% survival (Fig. 7D). However, RBC-NPs given intravenously did not play a protective role to mice, mice in this group all died within 8 days.

In another groups of mice, blood samples were taken for analysis 6 h post-infection (Fig. 7A). Compared to the negative control group of mice, WBC counts in ETX-infected mice were elevated 300%. The WBC counts of mice treated with RBC-NPs in lungs were closer to the negative control group of mice, but the WBC counts of mice treated with RBC-NPs intravenously were closer to those of mice in the positive control group. The increase in WBCs was mainly caused by 600% increases in NEU (Fig. 7E–F). This result showed that only RBC-NPs in lungs effectively attenuate the inflammatory response triggered in vivo by the ETX challenge in lungs. As before, we also tested blood markers of liver and kidney damage. The livers of mice were not damaged during the ETX challenge in lungs, but kidneys, the most sensitive organ to ETX, were slightly damaged with ETX challenge to lungs. Compared with injection of MNPs via veins, RBC-NPs in lungs appear to play a critical role in protecting kidneys of mice (Fig. 7G).

As with our experiment testing therapeutic efficacy injecting intravenously, we again dissected the main organs and stained tissue with H&E (Fig. 7B) for aerosol-challenged mice. Histopathological analysis showed that the brains of mice had no obvious pathological changes (Fig. 7H, Additional file 1: Fig. S11). The lungs of mice in the positive control group had serious damage, mainly manifested as congestion and edema, while the liver and kidneys of these mice had mild damage, mainly manifested as edema. However, these symptoms were significantly relieved by RBC-NPs in lungs.

To verify why pulmonary infection with ETX causes less damage to organs other than the lungs, and how RBC-NPs in lungs can protect mice, Cy5.5-ETX and DiR-RNPs were used to measure their interactions and metabolism in lungs (Fig. 7C). Quantitative data of fluorescence images in vitro of the DiR indicated that DiR-RNPs cannot escape the lungs (Fig. 7I). Quantitative data of fluorescence images in vitro of the Cy5.5 of the mice in the positive control group indicated that most Cy5.5-ETX remains in the lungs after 24 h, but after 48 h, some Cy5.5-ETX was detected in the livers and a smaller amount of Cy5.5-ETX was detected in the kidneys (Fig. 7J). This result showed why GST-ETX causes little damage to other organs when administered via the lung. However, for mice in the treatment group all Cy5.5-ETX was in the lungs and the distribution of the signal of GST-ETX was consistent with that of RBC-NPs. Thus, GST-ETX was completely neutralized in the lungs by RBC-NPs, preventing it from causing damage to the lungs and also preventing it from spreading beyond the lungs. This is how RBC-NPs protects the host against an ETX challenge to the lungs. This experiment demonstrated the efficacy of using RBC-NPs in lungs to treat of GST-ETX infection in lungs in vivo.

### Sustained protection of MNPs in vivo

**Fig. 8:**
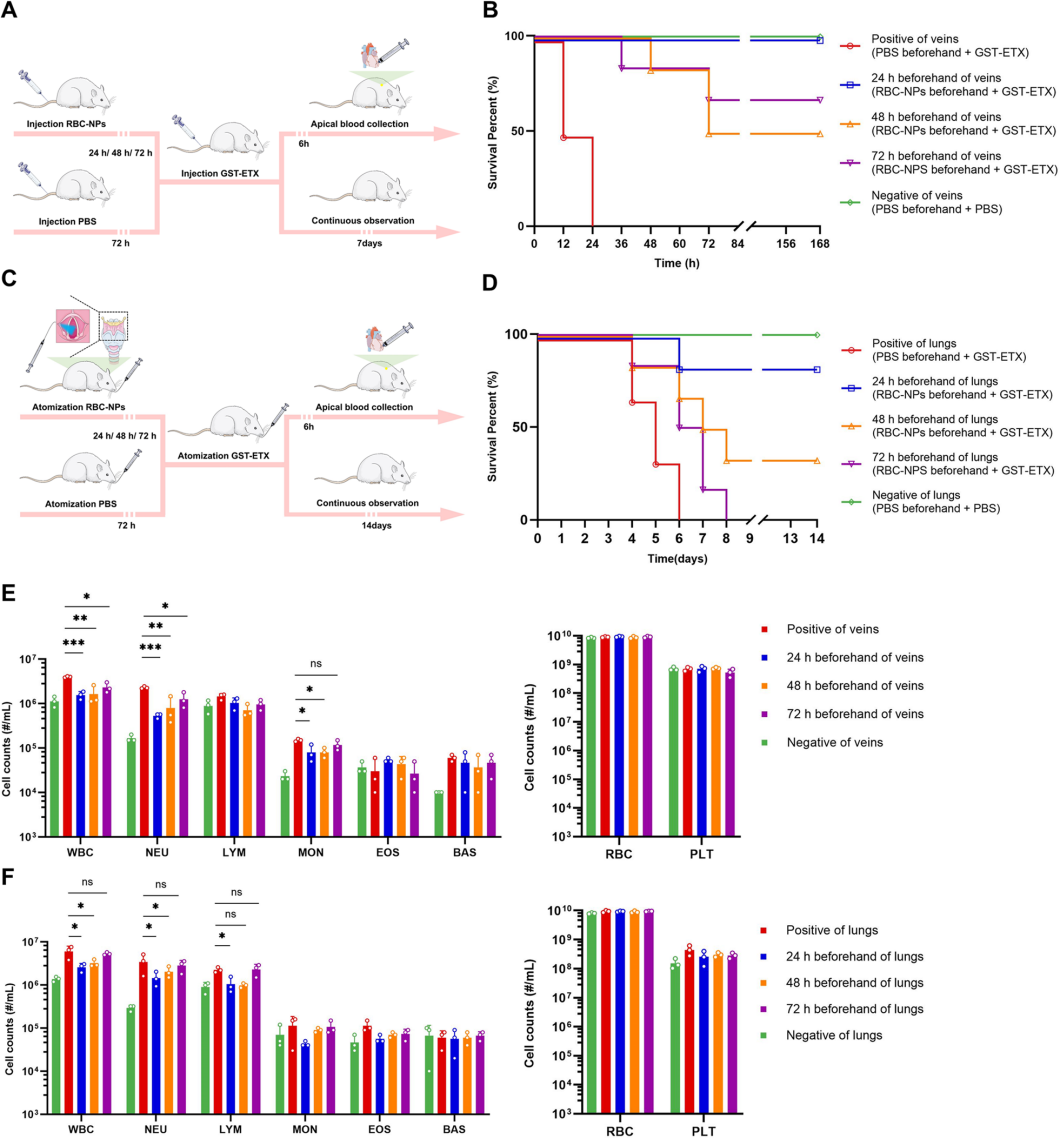
In vivo evaluation of projection provided by preinjected RBC-NPs. **A** Schematic illustration of the experiments. Three treatment groups of mice were injected with 2 mg RBC-NPs intravenously 24, 48, and 72 h prior to injection with 20 ng GST-ETX intravenously. The negative and positive control groups of mice were injected with PBS intravenously in the 72 h prior to toxin challenge, and mice were injected with 20 ng GST-ETX or PBS intravenously on the last day. **B** Survival curves of the mice for 7 days post-intravenous toxin challenge (n = 6). **C** Schematic illustration of the experiments. Three treatment groups of mice were injected with 2 mg RBC-NPs via aerosol into the lungs at 24, 48, and 72 h prior to aerosol challenge with 50 ng GST-ETX. The negative and positive control groups of mice had PBS via aerosol in the 72 h prior to aerosol lung exposure with 20 ng GST-ETX or PBS on the last day. **D** Survival curves of the mice for 7 days after aerosol toxin challenge (n = 6). **E** Blood cell counts of cells of mice injected intravenously (n = 3). **F** Blood cell counts of mice injected via aerosol into lungs (n = 3). Data are presented as mean ± SD. *p* < 0.05 (*), *p* < 0.01 (**), *p* < 0.001 (***), *p* ≥ 0.05 (ns)

Previous tissue in vitro imaging experiments had shown that RBC-NPs have a significantly longer circulation time than expected in vivo. This suggests that the stability of RBC-NPs can provides long-term protection in vivo. To test this, we delivered RBC-NPs to mice 1–3 days in advance of a toxin challenge via intravenous and aerosol, and then delivered GST-ETX to mice using the same delivery system. Mice were then observed for survival (Fig. 8A, C). RBC-NPs given intravenously 3 days in advance still provided protection for mice against ETX, and RBC-NPs in lungs 2 days in advance provided partial protection for mice (Fig. 8B, D). In other groups of mice, 6 h after completing toxin delivery, blood was taken for analysis (Fig. 8A, C). Blood biochemical analysis shows that, according to the two standards of WBC and NEU counts, appropriate early injection of RBC-NPs can provide protection to the host, although the protective effect decreases with time (Fig. 8E, F). This experiment demonstrated the efficacy of using RBC-NPs in advance to treat of GST-ETX infection in vivo.

## Discussion

As a potently toxic potential biowarfare or bioterrorism agent [1], ETX may represent a threat to human health, national security and animal husbandry economy for which no effective therapeutic drug is currently available. Inspired by the fact that ETX can form pores on cells membrane [3, 10] and based on the potential capabilities of the membrane-camouflaged biomimetic approach [22, 37], we explored whether MNPs might provide a medical countermeasure against the toxicity of ETX. Based on physical characteristics of MNPs, including ultra-small volume and higher specific surface area, as well as ability to interact with biomolecules and diffusion properties, it is expected that MNPs would exhibit stronger toxin binding ability than typical cells [38, 39]. As the results of in vitro evaluations of MNPs shown, MNPs have a more potent ability to bind to ETX than MDCK cells and human erythrocytes, indicating that MNPs has advantages over both cell lines and endogenous cells in binding to ETX. We hypothesize that MNPs can bind ETX faster than host cells in vivo. Subsequent experimental results confirmed our hypothesis, as RBC-NPs administered to mice can bind free ETX in the blood faster than the organs of the mice, resulting in significant reduction of inflammation and organ damage.

In addition, in this study, we assessed the neutralization performance and safety of MNPs against GST-ETX in vitro and in vivo, assessed the therapeutic efficacy of MNPs against GST-ETX delivered via different routes simultaneously and in advance in vivo, and verified the ways in which MNPs protect organs.

Membrane origins affect the neutralization capacity and safety of MNPs. Many cell lines that have been shown to be sensitive to ETX. However, the MDCK cell line is the most sensitive to ETX [40]. For animal RBCs, only human RBCs are sensitive to ETX [10]. Moreover, the morphological effects of ETX-induced hemolysis in RBCs resembles that in MDCK cells, but MDCK is much more sensitive to ETX. Therefore, we evaluated the neutralization capacity of MNPs camouflaged using these two unique cells membrane separately. MNPs replicate the ability of membrane-derived cells to bind to ETX, and likewise replicate the difference in sensitivity of the two kinds of cells to ETX; as such, MDCK-NPs are more effective than RBC-NPs at binding ETX [10]. However, the more capable MDCK-NPs performed worse in safety evaluations with mice. Comparing mice after being injected with RBC-NPs, MDCK-NPs and N2a cell membranes, we found that the source of the cells and the sophistication of cells affected the safety of MNPs. We demonstrated that three kinds of exogenous cell membranes do not cause liver or kidney damage in mice. However, because karyocytes have more complex cells membrane, MDCK cells membrane and N2a cells membrane cause a stronger immune response. This also reflects the advantages of human mature RBCs membrane. Karyocytes have more sophisticated biological functions than RBCs, and the biological characteristics of their membrane surface are also more sophisticated. In addition, other membrane proteins including C8-binding protein (C8bp), homologous restriction protein (HRP), decay-accelerating factor (DAF), membrane cofactor protein (MCP), complement receptor 1 (CR1), and CD59 on RBC surfaces fend off the attack by the complement system[41]. Thus, RBC-NPs have lower immunogenicity for reduced immune rejection of the host. For karyocytes such as MDCK cells and N2a cells, the more distantly related the membrane-derived cells are to the host, the more severe the immune response that is caused. MDCK cells line originate from normal kidney cells of a cocker spaniel dog, which is distantly related to murine species [35]; as a result, MDCK-NPs caused severer hypersensitivity reactions and led to the death of some mice. This suggests that the sophistication of the cell membrane is related to the in vivo safety of MNPs, and whether the membrane derived organism is homologous with the host will also influence in vivo safety. Hence, we suggest that the selection of cell membrane is also an important aspect for follow-up studies of MNPs. Under the premise that the desired therapeutic effect can be achieved, the cell membrane chosen should be as simple as possible. When a choice must be made between karyocytes, the cell membrane more closely related to the host is preferred.

MNPs alter the biological distribution of ETX among organs in the body. ETX was captured, neutralized and slowly delivered to the liver and spleen, where nanoparticles with ETX were phagocytized and metabolized. And the interaction between RBC-NPs and ETX, as well as metabolism of RBC-NPs and ETX complexes in vivo were verified for the first time in this study. This allowed us to visualize the process by which RBC-NPs treat ETX infection and protect organs in vivo. In our findings, RBC-NPs can neutralize toxins in the host within 5 min, and ETX and RBC-NPs remain combined in vivo, which ensures that RBC-NPs with neutralized ETX can be safely and stably be decomposed by liver and spleen. The liver and spleen are major a part of the mononuclear phagocyte system (MPS), and the majority of the injected nanoparticles are cleared from the bloodstream by cells of the MPS [42]. Biodistribution studies have shown this to be the case for all types of nanomaterials—micelles [43, 44], quantum dots [45, 46], gold nanoparticles [47, 48], and carbon nanotubes [49, 50]. Similarly, MNPs has the same distribution characteristics [20]. This phenomenon has been reported many times and is also the focus of research on nanoparticles in targeted therapy. However, for protecting host from fatal ETX challenge caused by intoxication with ETX, the function of MPS capture and decomposition can ensure that toxins are removed along with RBC-NPs, realizing the safe removal of toxins in the body. The residue after 24 h of most nanoparticles is negligible in the blood of mice [51–53]. Nonetheless, the RBC-NPs exhibit superior in vivo residence time [20]. In our study, RBC-NPs that neutralize toxins can achieve long circulation, and membrane-bound ETX on the RBC-NPs surface are stable. After 72 h of injection, the fluorescence level in the blood dropped by about 70%, while the fluorescence signal in the liver and kidney was relatively stable. Therefore, RBC-NPs bound to ETX in the blood are slowly captured by the MPS and continuously broken down, without a large accumulation in the MPS. This elucidates how the MNPs protect host organs in toxin challenges in vivo. Long circulation times that rely on RBC-NPs, can not only reduce the pressure on the MPS while ensuring the continuous decomposition of RBC-NPs and ETX, but also protects the host from an ETX challenge continuously, thereby prolonging the required injection cycle of RBC-NPs.

Pulmonary inhalation MNPs are as safe as intravenous injection MNPs [54]. We demonstrate that nebulized inhalation is a safe way to deliver MNPs into the lung. Both modalities can effectively protect mice against ETX. The blood markers of liver and kidney damage following lung delivery of GST-TEX were compared with intravenous injection GST-ETX and revealed differences in the degree of organ damage in mice after infection with toxin via different delivery methods]. Histopathological analysis showed that only the lungs of mice infected with ETX by aerosol had severe damage and that other organs had minor or no damage. Quantitative data of fluorescence images in vitro of the Cy5.5-ETX indicated that ETX cannot immediately escape the lungs, explaining the severe damage to the lungs by aerosol-infection with ETX. The fatal damage occurred in the lungs of mice, and lung delivery of MNPs can protect the lungs of mice faster and more directly than intravenous injection of MNPs. The results also indicate that pulmonary delivery of nanoparticles can more effectively treat the challenge of lung-inhaled toxins than intravenous injection of nanoparticles. This is important because toxin attacks by aerosols are likely in terrorist attacks. This is the first report of the use of RBC-NPs to neutralize inhaled toxins in the lung, and that this mode of infection is ineffectively treated by intravenous treatment. The results of this research can also provide an important reference for treating other aerosol-borne diseases, such as corona virus disease 2019 (COVID-19), which can cause lung damage and pulmonary anthrax, which can produce toxins in the lungs [55–58]. That RBC-NPs nebulized and delivered to the lungs can safely act as a protective effect has clear significance for studies of treatment in aerosol-borne diseases.

Our experiments also showed that, although RBC-NPs in the blood dropped rapidly on the first day, it remained detectable in the blood at 72 h and that RBC-NPs inhaled into the lungs remain present in the lungs for more than 72 h. The half-life of the fluorescence signal of RBC-NPs in the blood is close to 48 h, and the fluorescence signal of RBC-NPs in the lungs is not significantly reduced at 72 h. We verified that the delivery of RBC-NPs to the host in advance via vein or lung, can still provide protective effects to the host in the short term. Thus, RBC-NPs provide long-term protection that is difficult for chemical drugs to achieve. This is promising for treating ETX infection with RBC-NPs, in scenarios such as biochemical warfare; injecting susceptible people with RBC-NPs in advance or delivery of nebulized RBC-NPs to their lungs can continue to provide reliable protection for several days.

Based on the high efficiency and long-term protective performance of RBC-NPs, we suggest that MNPs have wide practicability in the treatment of infections. Toxins normally act on the plasma membrane or in cytoplasm of target cells, they must therefore interact with a membrane at some point [59]. Thus, by screening cell membranes and designing MNPs rationally, membrane-camouflaged biomimetic approaches have the potential to serve as a therapeutic platform for any toxin. To develop MNPs as nanomedicines, all that is required is understanding which cells are sensitive to the toxin, or the mechanism of action of the toxin. In the future, mixing cell membrane-camouflaged nanoparticles or mixing different MNPs could become a multifunctional and powerful therapeutic platform for treating toxins. Most importantly, this therapeutic platform can treat both acute toxin infections and provide long-term protection against toxins.

## Conclusion

In this study, a nanomedicine was developed based on membrane-camouflaged biomimetic approaches, to treat fatal infection caused by ETX. The safety and therapeutic efficacy of MNPs was assessed in vitro and in vivo*,* and was used as a reference for screening membranes of cells. RBC-NPs can neutralize ETX efficiently in vitro and in vivo. Only the membrane from RBCs is used to camouflage nanoparticles that protect mice from ETX infection, as the RBC membrane is superior in safety. Nebulized inhalation and intravenous injection with RBC-NPs both safety treat ETX infection in corresponding modes of infection. RBC-NPs injected in the vein can protect organs by altering the biological distribution of ETX. Inoculation with RBC-NPs in over a period up to 3 days in advance can provide protection for the host in a time-dependent manner. Thus, our experimental results suggest RBC-NPs provide a unique and feasible nanomedicine against ETX infection.

## Materials and methods

### Materials

The PLGA were purchased from LACTEL Absorbable Polymers (Brimingham, USA). The 100 nm polycarbonate membranes were purchased from Avanti Polar Lipids (Alabama, USA). The GST-ETX monoclonal antibody was developed previously by colleagues in our laboratory. MDCK cells were preserved previously by colleagues in our laboratory. Mouse neuroblastoma N2a cells were purchased from BeNa Culture Collection (Beijing, China). The MTS were purchased from Promega Corporation (Madison, USA). BABL/c mice of SPF grade were purchased from Sipeifu (Beijing, China). Cy5.5-antibody conjugation kits were purchased from Bioss (Beijing, China). DiR were purchased from Invitrogen (Carlsbad, USA).

### Preparation of MNPs

To begin with, PLGA nanoparticles with a diameter of about 100 nm were prepared by using the emulsion-evaporation method. Carboxy-terminated PLGA was dissolved in dichloromethane (DCM) at 0.67 dL/g and mixed thoroughly with polyvinyl alcohol (PVA) aqueous solution. The mixture was mixed into a homogeneous emulsified state by a Qsonica Q125 (Qsonica LLC, USA) sonicator and then stirred in fume hoods for 4 h at 25 °C by magnetic stirrers. After removing the DCM in the mixture, PLGA nanoparticles were obtained using low temperature ultrahigh speed centrifugation followed by washing three times using ultrapure water to remove the residual PVA. Finally, the PLGA nanoparticles suspension was freeze-dried to obtain dried PLGA nanoparticles.

RBCs were obtained by removing the upper plasma from 5 mL of whole blood that had been centrifuged at 1,000 × g at 4 °C for 10 min. Next, 1 mL of collected RBCs were washed three times using 1 × PBS and then resuspended in 2 mL of ultrapure water. Osmotic pressure was used to break the RBCs, and then broken erythrocyte membranes were washed three times using 1 × PBS to remove residual hemoglobin (HGB). Membranes were resuspended in 1 mL of 1 × PBS and mixed with 10 mg PLGA nanoparticles. The mixture was then extruded through 100 nm polycarbonate membranes to prepare RBC-NPs.

MDCK cells were cultured with Dulbecco's Modified Eagle Medium (DMEM) supplemented with 10% fetal bovine serum (FBS). Using the same method as above for RBCs to extract membranes, membranes from 1 × 10^8^ cells were resuspend in 1 mL of 1 × PBS and mixed with 10 mg PLGA nanoparticles. The mixture was then extruded through 100 nm polycarbonate membranes to prepare MDCK-NPs.

### Characterization of nanoparticles and MNPs

The Flow NanoAnalyzer (NanoFCM, China) was used to measure the diameter and size distribution of the nanoparticles at 0.01 mg/mL in ultrapure water. The Zetasizer Nano ZS90 (Malvern, UK) was used to measure the PDI and zeta potentials of the nanoparticles were determined at 0.01 mg/mL in ultrapure water and at 25 °C. Transmission electron microscopy images from the FEI Tecnai G2 F30 (FEI, US) were used to characterize morphology of the nanoparticles.

### Cell culture

MDCK cells and N2a cells were cultured in DMEM with 10% serum at 37 °C in a 5% CO_2_ atmosphere and 100% humidity. The cells were used for the experiments within the first 20 passages.

### Cytotoxicity Assay

Cytotoxic activity was measured by MTS colorimetric assay with MDCK cells. MDCK cells were spread into 96-well plates at a cell density of 1 × 10^5^ cells/mL and incubated at 37 °C for 24 h. The solution to be measured was added at a series of dilutions concentrations and incubated for 1 h at 37 °C in 5% CO_2_ atmosphere. Next, the culture medium was removed, and plates washed with PBS three times. MTS was added to plate wells, incubated for 3 h, and then toxicity estimated by measuring absorbance at 492 nm. All experiments were conducted in triplicate.

### High-content Imaging

ImageXpress and MetaXpress (Molecular Devices, USA) were used to detect kinetic changes in MDCK cells. MDCK cells were incubated with GST-ETX, MDCK-NPs and an equal amount of 4′, 6-diamidino-2-phenylindole (DAPI) (5 μg/ mL) for 1 h, then the culture medium was removed, and cells washed with PBS three times to remove excess DAPI. Next, DAPI and propidium iodide (PI) were added into the MDCK cells’ culture medium and incubated for 3 h. After incubation, fluorescence signals were observed under the confocal microscope.

### Animals

BABL/c mice of SPF grade were further bred in the accredited animal facility of the Experiment Center of the Academy of Military Medical Sciences. Mice were used at 6–10 weeks of age. The number of animals in each group was set according to statistical verification and previous studies.

### Nanoparticles and GST-ETX Fluorescent staining

The Cy5.5-antibody conjugation kit (Beijing, China) and DiR (Carlsbad, USA) were used to label the nanoparticles and GST-ETX separately.

### In vitro imaging of major tissues

Dyed nanoparticles and ETX were injected into 6–8-week-old healthy female BABL/c mice. At 5 min, 24 h, 48 h, and 72 h post-injection, blood and major organ samples (liver, heart, spleen, lung, kidney and brain) were collected. Nanoparticles and GST-ETX distribution were detected by IVIS Spectrum (PerkinElmer, USA) imaging system.

### Enzyme-linked immunosorbent assay (ELISA)

We used the mouse IgE ELISA kit (Beyotime, China) to detect the IgE of mice, following the manufacturer’s instructions. All assays were performed in triplicate. Optical density was measured at a wavelength of 450 nm with a spectrophotometer.

### Histopathological analysis of samples

Samples were collected after injection with GST-ETX (800 ng/kg) and nanoparticles, then fixed in 4% formaldehyde solution. The histopathological analysis of samples was performed by H&E staining.

### Statistical analysis

All data are expressed as mean ± SD. Statistical analysis was performed using GraphPad Prism. Statistical comparisons were performed using Student’s *t*-tests, one-way ANOVAs, or two-way ANOVAs. Statistical significance was indicated by *p-*values < 0.05 (*), < 0.01 (**), or < 0.001 (***); non-significant differences were indicated by *p-* values ≥ 0.05 (ns).

## Supplementary Information

**Additional file 1:** Systematic evaluation of membrane-camouflaged nanoparticles in neutralizing *Clostridium perfringens* ε-toxin. ** Figure S1**. In vitro toxicities of recombinant ETX. ETX with different tags (GST and 6×His) did not significantly differ in toxicities (n = 3). Data are presented as the means ± SD. **Figure S2**. MDCK cells were exposed to 2 mg nanoparticles and 20 nM of GST-ETX for 1h at 37°C. The cells were observed by confocal microscopy. (Scale bar: 1 mm). **Figure S3**. Four groups of eight-week-old female BALB/c mice, were injected with increasing dosages of GST-ETX in intravenous respectively. The survival curves of the mice in the next 7 days (n = 6). **Figure S4**. Representative sections made from various organs of experimental mice with intravenous injection, stained with H&E (scale bar: 2 mm). **Figure S5**. In vitro fluorescence images of DiR in organs of mice which injected intravenously with Cy5.5-ETX and PBS. **Figure S6**. In vitro fluorescence images of DiR in organs of mice which injected intravenously with Cy5.5-ETX and DiR-RNPs. **Figure S8**. In vitro fluorescence images of Cy5.5 in organs of mice which injected intravenously with Cy5.5-ETX and PBS. Figure S9. In vitro fluorescence images of Cy5.5 in organs of mice which injected intravenously with Cy5.5-ETX and DiR-RNPs. **Figure S9**. Real-time in vivo fluorescence images of mice after lung delivery or intravenous injection 10 min. (A) Lung delivery DiR-RNPs, DiR-RNPs were evenly dispersed in lung of the mouse but did not escape the lung. Radiant efficiency exceeded 2×109 in the lung. (B) Lung delivery PBS. (C) Intravenous injection PBS. (D) Intravenous injection DiR-RNPs, DiR-RNPs spread throughout the mouse by the bloodstream. The systemic radiation efficiency of mouse was generally low, and the maximum radiation efficiency did not reach 2×109. **Figure S10**. Four groups of eight-week-old female BALB/c mice, were introduced by aerosol into the lungs with increasing dosages of GST-ETX. The survival curves of the mice in the next 14 days (n = 6). **Figure S11**. Representative sections made from various organs of experimental mice with lung delivery, stained with H&E (scale bar: 2 mm).

## Data Availability

Data from this study are available within the article and its Supplementary Information files, or from the corresponding author upon reasonable request.

## Abbreviations

ALP: Alkaline phosphatase
Bare NPs: Bare nanoparticles
BAS: Basophil
Ca: Serum calcium
Cy5.5: Cyanine 5.5
Cy5.5-ETX: ETX which was label by Cy5.5
DAPI: 4′, 6-Diamidino-2-phenylindole
DiR: DiOC18(7)
DiR-RNPs: RBC-NPs which was label by DiR
DLS: Dynamic light scattering
DMEM: Dulbecco's modified eagle medium
EOS: Eosinophil
ETX: *Clostridium perfringens* ε-toxin
GLU: Glucose
GST: Glutathione-S-transferase
H&E: Hematoxylin and eosin
MDCK cells: Madin-Darby canine kidney cells
MDCK-NPs: MDCK cells membrane-camouflaged nanoparticles
MNPs: Membrane-camouflaged nanoparticles
MPS: Mononuclear phagocyte system
Na: Serum sodium
NEU: Neutrophils
N2a cells: Mouse neuroblastoma N2a cells
PDI: Polydispersity index
PI: Propidium iodide
PLGA: 50:50 Poly (DL-lactide-co-glycolide) Carboxylate End Group
PLT: Platelet
RBCs: Red blood cells
RBC-NPs: Red blood cells membrane-camouflaged nanoparticles
WBC: White blood cell
