# Supplementary material for: Systematic evaluation of membrane-camouflaged nanoparticles in neutralizing Clostridium perfringens ε-toxin
Source: J Nanobiotechnology. 2023 Mar 17;21:95. doi: 10.1186/s12951-023-01852-z (PMC10021051; doi:10.1186/s12951-023-01852-z)
Supplement: Supplementary file 1 — Additional file 1: Systematic evaluation of membrane-camouflaged nanoparticles in neutralizing Clostridium perfringens ε-toxin. Figure S1. In vitro toxicities of recombinant ETX. ETX with different tags (GST and 6×His) did not significantly differ in toxicities (n = 3). Data are presented as the means ± SD. Figure S2. MDCK cells were exposed to 2 mg nanoparticles and 20 nM of GST-ETX for 1h at 37°C. The cells were observed by confocal microscopy. (Scale bar: 1 mm). Figure S3. Four groups of eight-week-old female BALB/c mice, were injected with increasing dosages of GST-ETX in intravenous respectively. The survival curves of the mice in the next 7 days (n = 6). Figure S4. Representative sections made from various organs of experimental mice with intravenous injection, stained with H&E (scale bar: 2 mm). Figure S5. In vitro fluorescence images of DiR in organs of mice which injected intravenously with Cy5.5-ETX and PBS. Figure S6. In vitro fluorescence images of DiR in organs of mice which injected intravenously with Cy5.5-ETX and DiR-RNPs. Figure S8. In vitro fluorescence images of Cy5.5 in organs of mice which injected intravenously with Cy5.5-ETX and PBS. Figure S9. In vitro fluorescence images of Cy5.5 in organs of mice which injected intravenously with Cy5.5-ETX and DiR-RNPs. Figure S9. Real-time in vivo fluorescence images of mice after lung delivery or intravenous injection 10 min. (A) Lung delivery DiR-RNPs, DiR-RNPs were evenly dispersed in lung of the mouse but did not escape the lung. Radiant efficiency exceeded 2×10^9^ in the lung. (B) Lung delivery PBS. (C) Intravenous injection PBS. (D) Intravenous injection DiR-RNPs, DiR-RNPs spread throughout the mouse by the bloodstream. The systemic radiation efficiency of mouse was generally low, and the maximum radiation efficiency did not reach 2×10^9^. Figure S10. Four groups of eight-week-old female BALB/c mice, were introduced by aerosol into the lungs with increasing dosages of GST-ETX. The survival curves of the [file 12951_2023_1852_MOESM1_ESM.docx]

**Additional Information for**

**New insights into the membrane-camouflaged nanoparticles for the treatment of ETX intoxication**

**This PDF file includes:**

1. **Figure S1** to **S11**


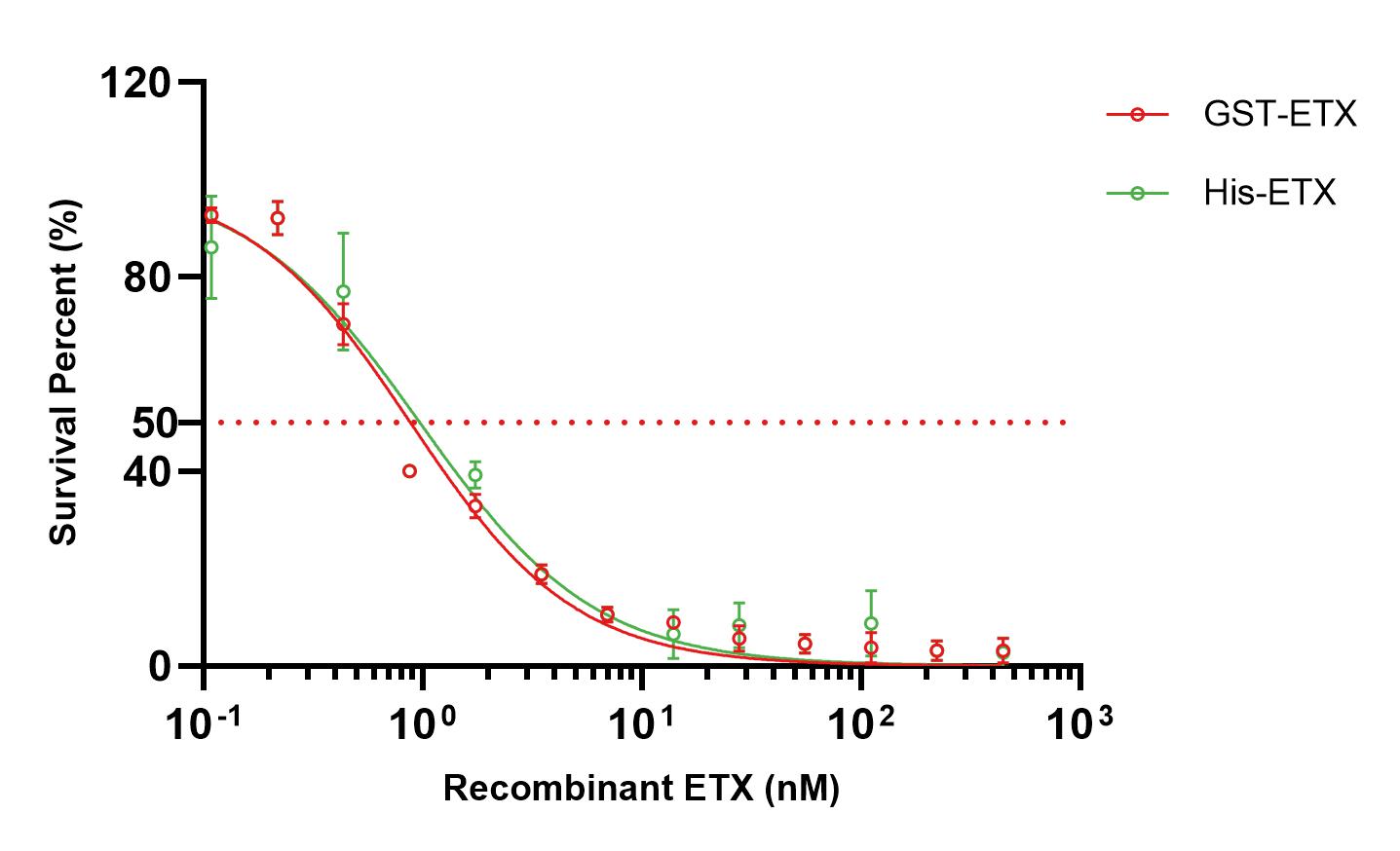


**Figure S1.** In vitro toxicities of recombinant ETX. ETX with different tags (GST and 6×His) did not significantly differ in toxicities (n=3). Data are presented as the means ± SD.


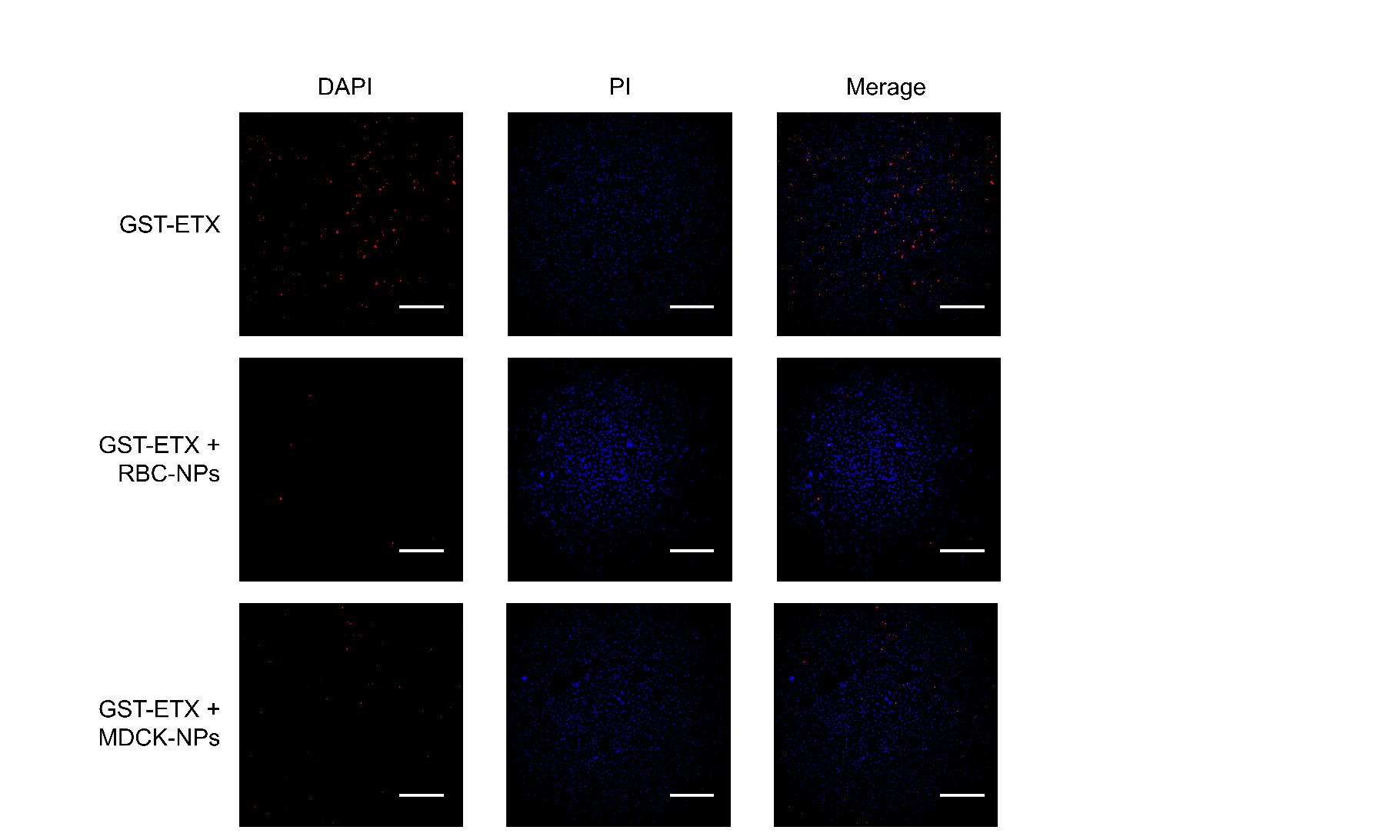


**Figure S2.** MDCK cells were exposed to 2 mg nanoparticles and 20 nM of GST-ETX for 1h at 37°C. The cells were observed by confocal microscopy. (Scale bar: 1 mm).


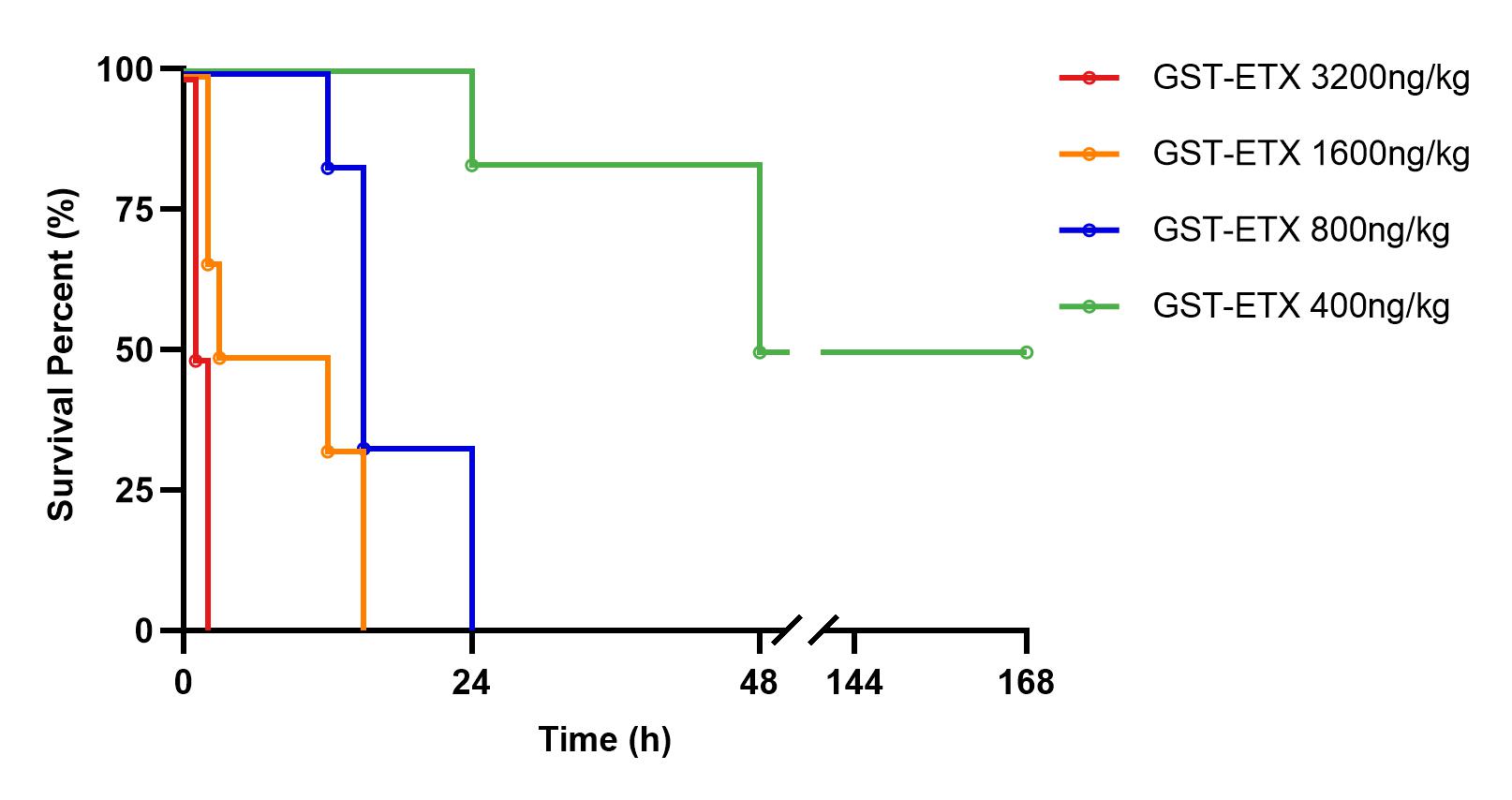


**Figure S3.** Four groups of eight-week-old female BALB/c mice, were injected with increasing dosages of GST-ETX in intravenous respectively. The survival curves of the mice in the next 7 days (n = 6).


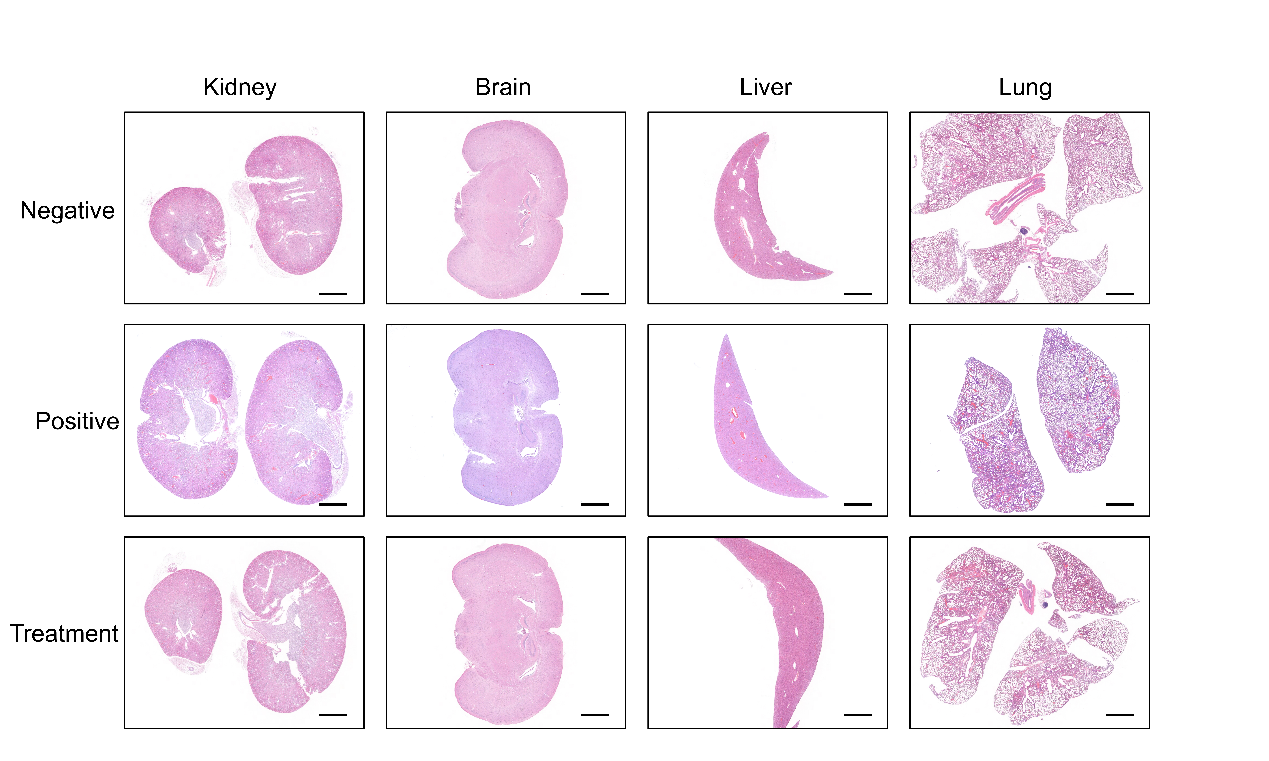


**Figure S4.** Representative sections made from various organs of experimental mice with intravenous injection, stained with H&E (scale bar: 2 mm).


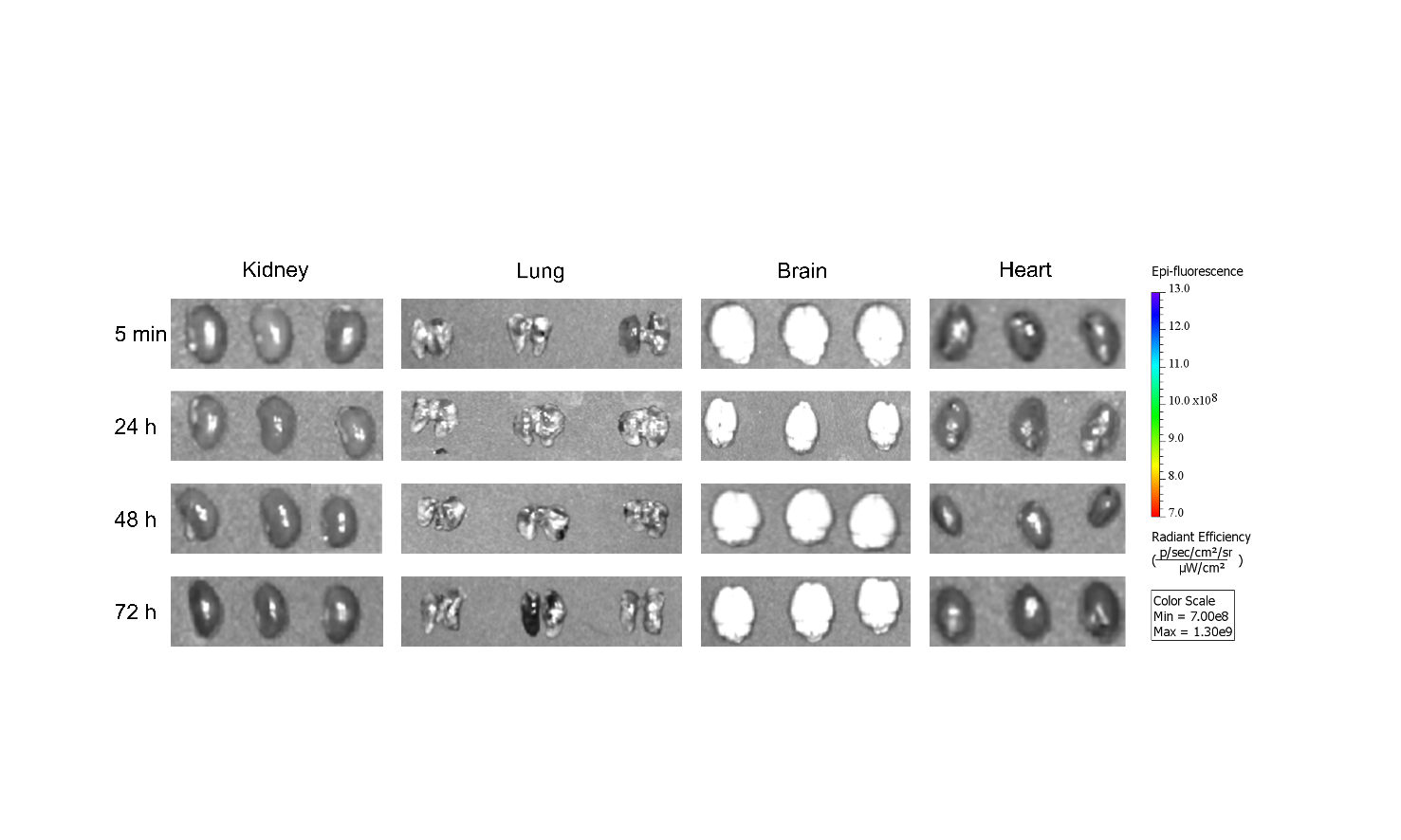


**Figure S5.** *In vitro* fluorescence images of DiR in organs of mice which injected intravenously with Cy5.5-ETX and PBS.


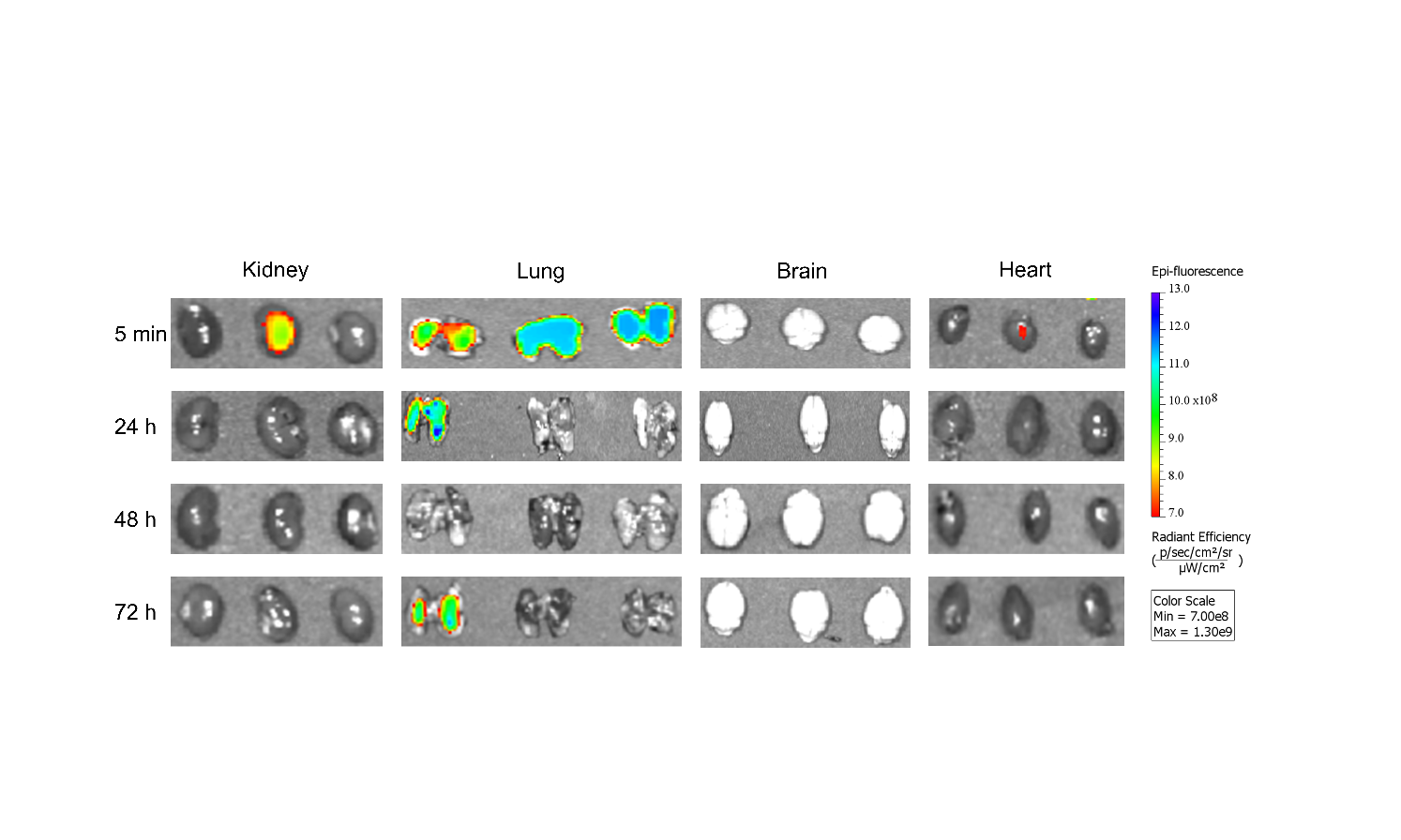


**Figure S6.** *In vitro* fluorescence images of DiR in organs of mice which injected intravenously with Cy5.5-ETX and DiR-RNPs.


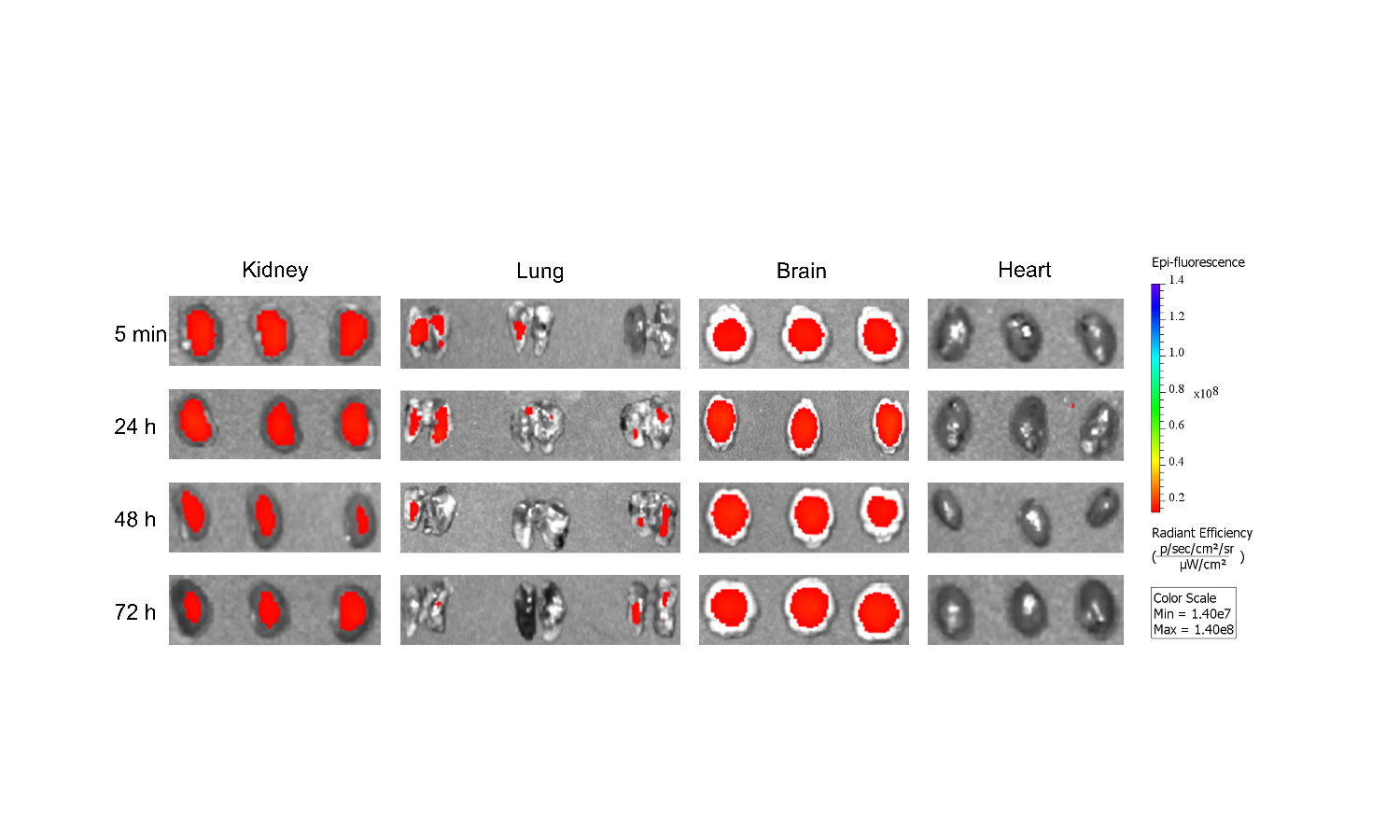


**Figure S8.** *In vitro* fluorescence images of Cy5.5 in organs of mice which injected intravenously with Cy5.5-ETX and PBS.


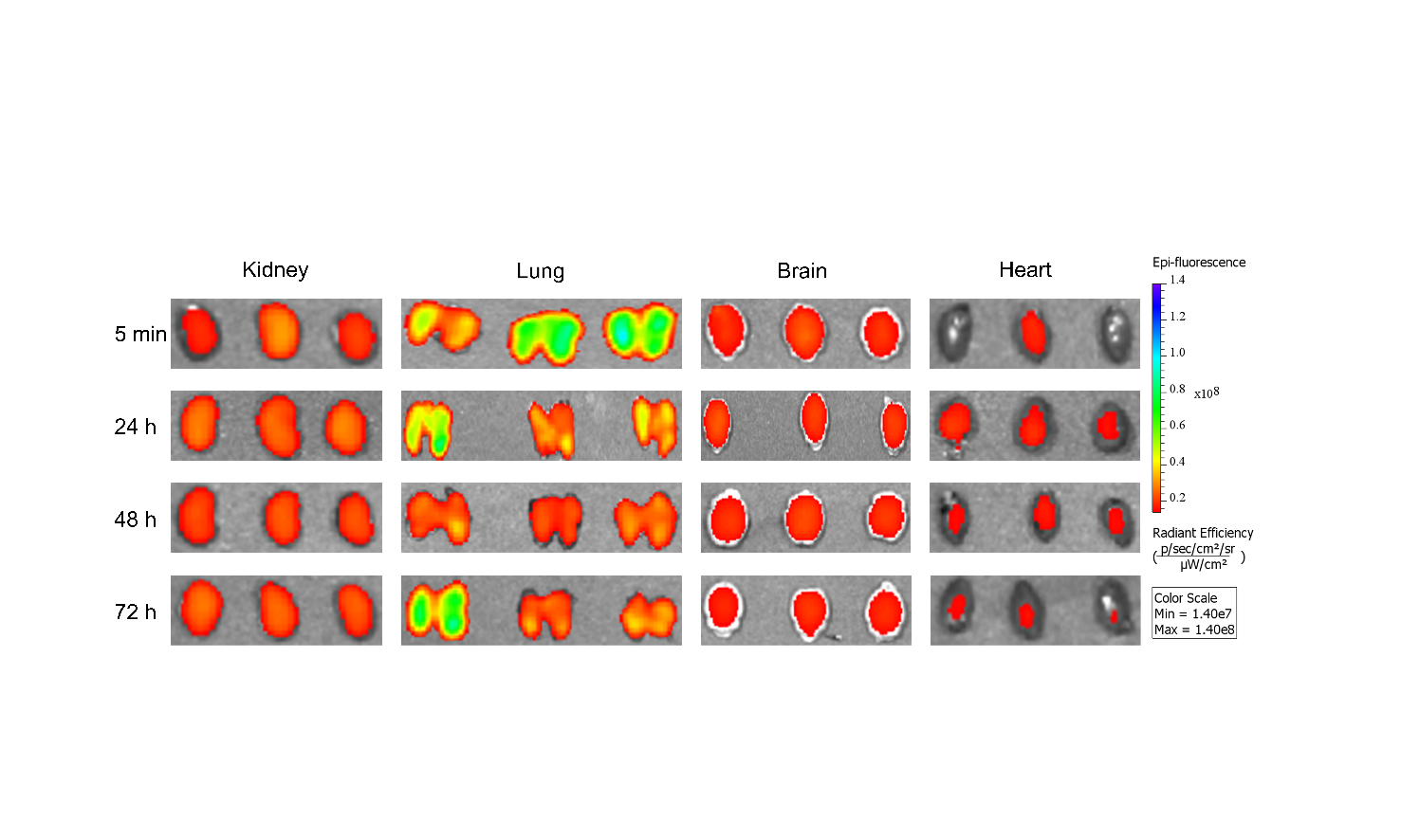


**Figure S9.** *In vitro* fluorescence images of Cy5.5 in organs of mice which injected intravenously with Cy5.5-ETX and DiR-RNPs.


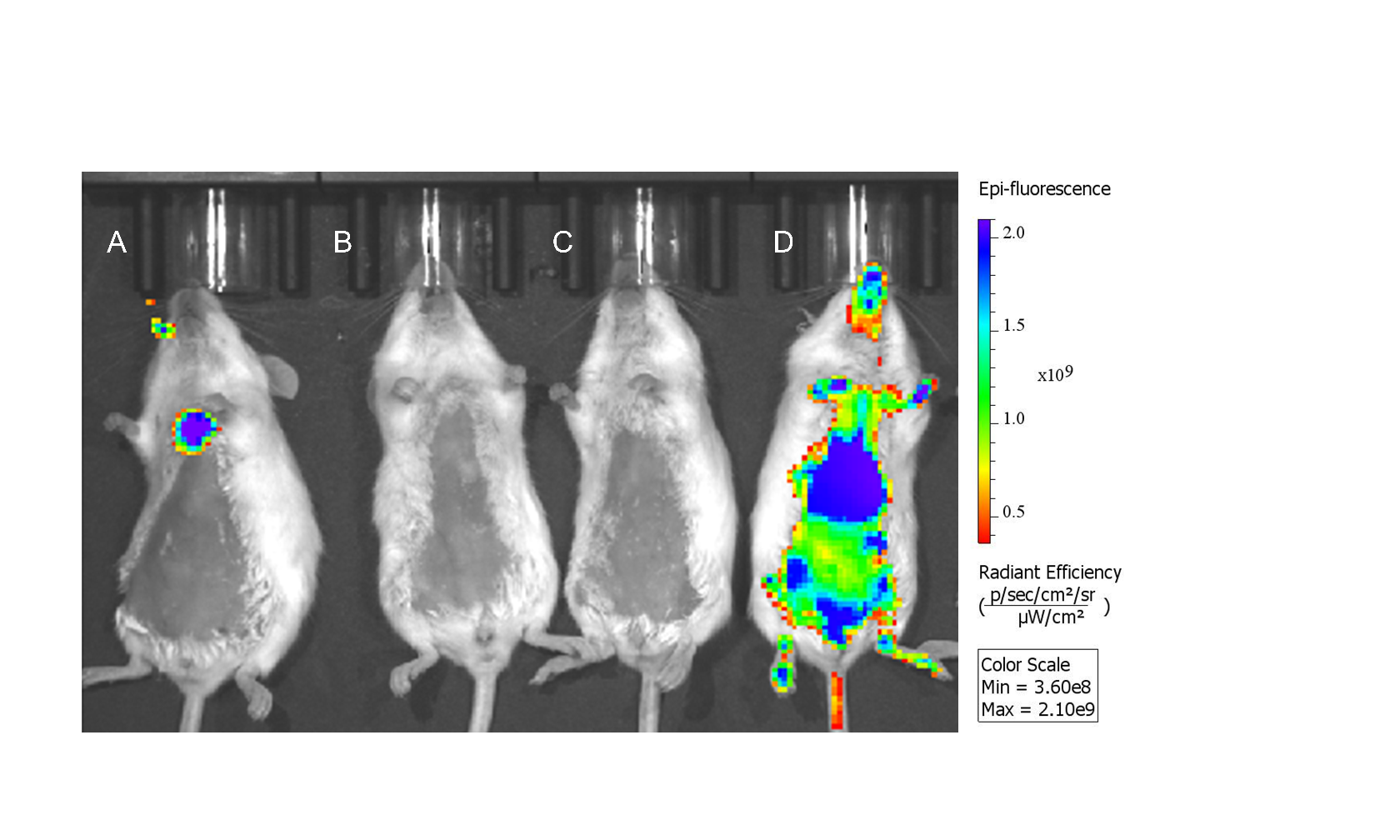


**Figure S9.** Real-time *in vivo* fluorescence images of mice after lung delivery or intravenous injection 10 min. (**A**) Lung delivery DiR-RNPs, DiR-RNPs were evenly dispersed in lung of the mouse but did not escape the lung. Radiant efficiency exceeded 2×10^9^ in the lung. (**B**) Lung delivery PBS. (**C**) Intravenous injection PBS. (**D**) Intravenous injection DiR-RNPs, DiR-RNPs spread throughout the mouse by the bloodstream. The systemic radiation efficiency of mouse was generally low, and the maximum radiation efficiency did not reach 2×10^9^.


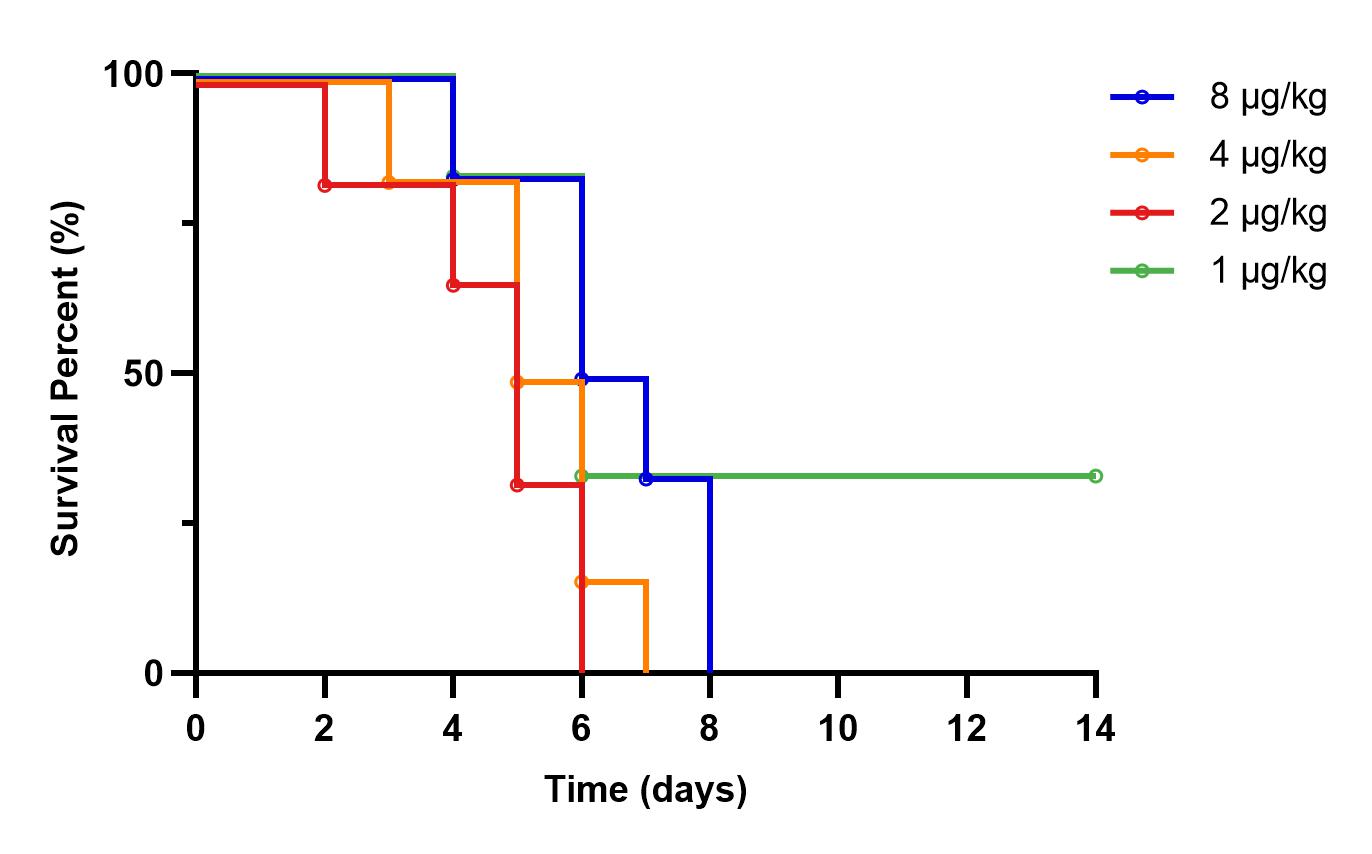


**Figure S10.** Four groups of eight-week-old female BALB/c mice, were introduced by aerosol into the lungs with increasing dosages of GST-ETX. The survival curves of the mice in the next 14 days (n = 6).


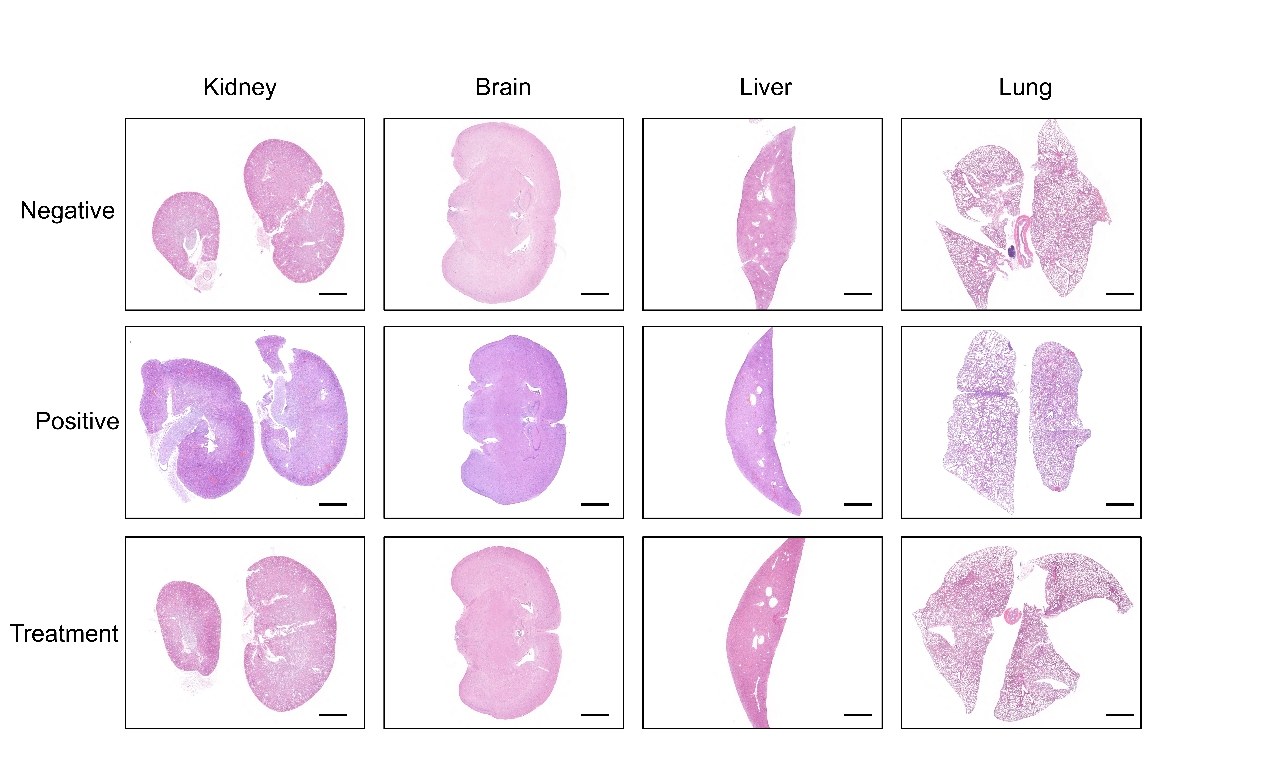


**Figure S11.** Representative sections made from various organs of experimental mice with lung delivery, stained with H&E (scale bar: 2 mm).
